# Natural images are reliably represented by sparse and variable populations of neurons in visual cortex

**DOI:** 10.1038/s41467-020-14645-x

**Published:** 2020-02-13

**Authors:** Takashi Yoshida, Kenichi Ohki

**Affiliations:** 1 Department of Physiology, The University of Tokyo School of Medicine, Tokyo, Japan; 2 Department of Molecular Physiology, Graduate School of Medical Sciences, Kyushu University, Fukuoka, Japan; 3 CREST, Japan Science and Technology Agency, Tokyo, Japan; 4 International Research Center for Neurointelligence (WPI-IRCN), The University of Tokyo, Hongo, Bunkyo-ku, Tokyo, 113-0033 Japan

**Keywords:** Computational biology and bioinformatics, Neuroscience, Sensory processing

## Abstract

Natural scenes sparsely activate neurons in the primary visual cortex (V1). However, how sparsely active neurons reliably represent complex natural images and how the information is optimally decoded from these representations have not been revealed. Using two-photon calcium imaging, we recorded visual responses to natural images from several hundred V1 neurons and reconstructed the images from neural activity in anesthetized and awake mice. A single natural image is linearly decodable from a surprisingly small number of highly responsive neurons, and the remaining neurons even degrade the decoding. Furthermore, these neurons reliably represent the image across trials, regardless of trial-to-trial response variability. Based on our results, diverse, partially overlapping receptive fields ensure sparse and reliable representation. We suggest that information is reliably represented while the corresponding neuronal patterns change across trials and collecting only the activity of highly responsive neurons is an optimal decoding strategy for the downstream neurons.

## Abstract (web summary)

Natural scenes sparsely activate V1 neurons. Here, the authors show that a small number of active cells reliably represent visual contents of a natural image across trials regardless of response variability, due to the diverse and partially overlapping representations of individual cells.

## Introduction

Sensory inputs sparsely activate neurons in the sensory cortex^1–9^, which is postulated to be an efficient information coding^4,10^. However, how sparsely active neurons represent information and how the information is optimally decoded from the sparse representation have not been determined.

In the primary visual cortex (V1), simple cells each have a receptive field (RF) structure that is modeled by a two-dimensional (2D) Gabor function^11^. Theoretically, a single natural scene is represented by a relatively small number of neurons using Gabor-like RFs, whereas information about multiple scenes is distributed among the neuronal population^10,12,13^. Indeed, V1 neurons sparsely respond to natural scenes^2,3,5–9,14^. Population activity with higher sparseness exhibits greater discriminability between natural scenes^5^.

What types of information from natural scenes are represented in the sparsely active neurons in the brain? The visual contents of natural scenes are reconstructed from single-unit activity within the lateral geniculate nucleus collected from several experiments^15^ and functional magnetic resonance imaging data from the visual cortices^16–19^. However, whether the visual contents of natural images could be represented by simultaneously recorded, sparsely active neurons has not been addressed experimentally. It also remains to be revealed which decoding strategy is the most optimal for sparse representation. Whether only the small number of strongly active neurons represent information, or whether the remaining neurons contribute additional information remains to be determined. Furthermore, do the sparsely active neurons reliably represent the natural image contents, regardless of trial-to-trial response variability? Although a computational model^20^ has suggested that sparse and overcomplete representation is optimal for natural image representation with unreliable neurons, this model has not been examined experimentally.

We also address how visual information is distributed in a neuronal population. Some neurons are “unresponsive” to simple visual stimuli, such as moving gratings (e.g., the response rate of the mouse V1 is 26–68%)^21–27^. However, this may be partly because visual stimuli are not complex enough to completely cover the RF properties of all neurons. The proportion of neurons involved in the information processing is a matter of debate^28,29^.

Here, we examined how sparsely active neurons represent natural image contents and how the information was optimally decoded from this sparse representation. Using two-photon calcium (Ca^2+^) imaging, we recorded visual responses to natural images from the V1 of anesthetized and passively viewing awake mice. A small percentage of neurons responded to each image, which was sparser than that predicted by a linear encoding model. On the other hand, most neurons were involved in processing of natural images. The visual contents of a natural image were linearly decodable from a small number of highly responsive neurons, and the additional use of the remaining neurons even degraded the reconstruction performance. The responsive neurons reliably represent the image, regardless of trial-to-trial response variability, which was supported by multiple neurons with partially overlapping representation. These results revealed a new representation of a natural image by a small number of neurons in which information is reliably represented, while the corresponding neuronal patterns change across trials and implies that collecting only the activity of highly responsive neurons is an optimal decoding strategy for sparse representation. The preliminary results of this study have been published in abstract form^30^ and on a preprint server^31^.

## Results

### Sparse visual responses to natural images

**Fig. 1:**
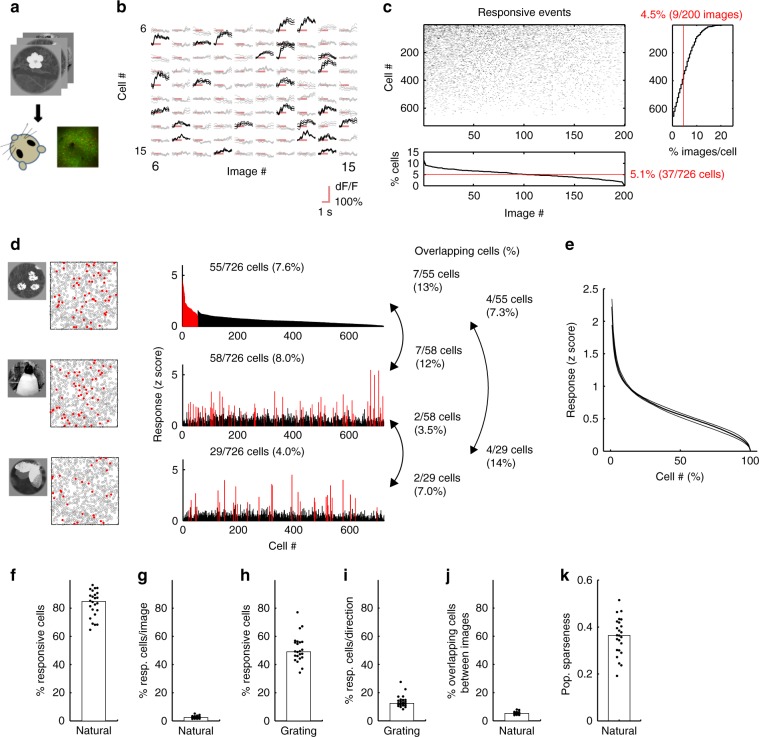
Sparse visual responses to natural images. **a** Experimental schematic. Natural images are presented, and the activity of V1 neurons was recorded using two-photon Ca^2+^ imaging. **b** Examples of trial-averaged visual responses. The three lines for each response indicate the mean and the mean ± S.E.M. Black: significant responses, gray: non-significant responses, and red: stimulus periods. **c** Significant response events in an example plane (upper left). Bottom: the percentage of responsive cells for each image. Right: the percentage of images to which each cell responded. Red lines (bottom and right) indicate median values. **d** Examples of population response patterns to three images. Left: stimulus images and the spatial distributions of cells in an imaging area (side length: 507 microns). The red-filled and gray open circles indicate the responsive and remaining cells, respectively. Right: histograms of the visual responses to images presented in the left panels. Cells are divided into responsive (red) and the remaining groups (black), and are sorted in each group by the response amplitude to the image presented in the top row. The cell number order is fixed among the three histograms. **e** Distribution of the amplitude of responses to single images. The cell # is sorted by the amplitude of the response to each image and averaged across images in a plane. After normalizing the cell # (*x*-axis), data were collected across planes. The median (thick line) and 25–75th percentiles (thin lines) are shown. **f** Percentages of visually responsive cells. **g** Percentages of responsive cells per image. **h** Percentages of responsive cells for the moving grating. **i** Percentages of responsive cells for each direction of the moving grating. **j** Percentages of overlap of responsive cells between the natural images. **k** Population sparseness. **f**–**k** Each dot indicates data obtained from one plane, and the medians across planes are shown as bars. **e**–**g**, **j**, **k** *N* = 24 planes. **h**, **i** *N* = 23 planes (one plane data were discarded because of FOV drift during imaging). The stimulus images in **a** and **d** are adapted from the databases in refs. ^56,57^ with permission. Source data are provided as a Source Data file.

We presented flashes of natural images as visual stimuli (200 images for main dataset, dataset 1; 1000–2000 images for another dataset, dataset 2). Each image was consecutively flashed three times in a trial (three 200 ms presentations interleaved with 200 ms of a gray screen) and presented at least 12 times for dataset 1 and three to eight times for dataset 2 in a recording session (Fig. 1a, Methods section). Using two-photon Ca^2+^ imaging, we recorded single neurons’ activity from anesthetized mouse V1 (560 (284–712) cells/plane, median (25–75th percentiles); *n* = 24 planes from 14 mice for dataset 1, *n* = 4 planes from three mice for dataset 2, 260–450 microns in depth; see Fig. 1b for representative response traces). Results described below were obtained from dataset 1 unless otherwise stated.

Most neurons in a local population were visually responsive. Responsive neurons were determined by one-way analysis of variance (ANOVA, *p* < 0.01; data with responses to 200 images and one baseline in each trial, see Methods section). Across planes, 85% (77–90%) of neurons were responsive (Fig. 1f). Using label-shuffled data, we found a 1.0% (0.9–1.0%) false positive rate. Thus, most neurons were involved in visual processing of natural images.

We next examined the percentage of neurons that responded to each image. For each responsive neuron determined by ANOVA, a significant response to each image was defined by a *t*-test and amplitude threshold (*p* < 0.01, using a *t*-test and >10% trial-averaged visual response; see Methods section and Supplementary Fig. 1). Figure 1c presents plots of significant visual response events for all images (*x*-axis) across all neurons (*y*-axis) in an example plane (*n* = 726 cells, depth: 360 microns from the brain surface). Across planes, 2.5% (1.8–3.0%) of neurons were responsive for each image (Fig. 1g, Supplementary Fig. 1; Fig. 1c bottom for the example case). This low response rate was not due to poor recording conditions. The same neurons responded well to moving gratings (49% (45–56%) of cells were responsive for at least one grating direction, and 12.5% (11–14.8%) responded to each direction of grating, Fig. 1h, i).

The responsive neurons only slightly overlapped between images. In examples for three natural images (Fig. 1d), each image activated different subsets of neurons that exhibited small overlaps between images (Fig. 1d, right column). Of the responsive cells, 5.4% (4.8–6.0%) exhibited overlap between two images (Fig. 1j). Furthermore, only a small number of neurons exhibited visual responses with large amplitudes, which is a property of sparse representations (Fig. 1e). The population sparseness^2,3^ was comparable to that from a previous report on mouse V1 (ref. ^5^; 0.36 (0.30–0.42), Fig. 1k, see Methods section). Together, each natural image activated a small number of neurons, whereas most neurons were visually responsive. The latter result represents the first report of the visual responsiveness of most neurons in mouse V1 to natural image stimuli^28,29^.

### Partially overlapping representations of visual features

**Fig. 2:**
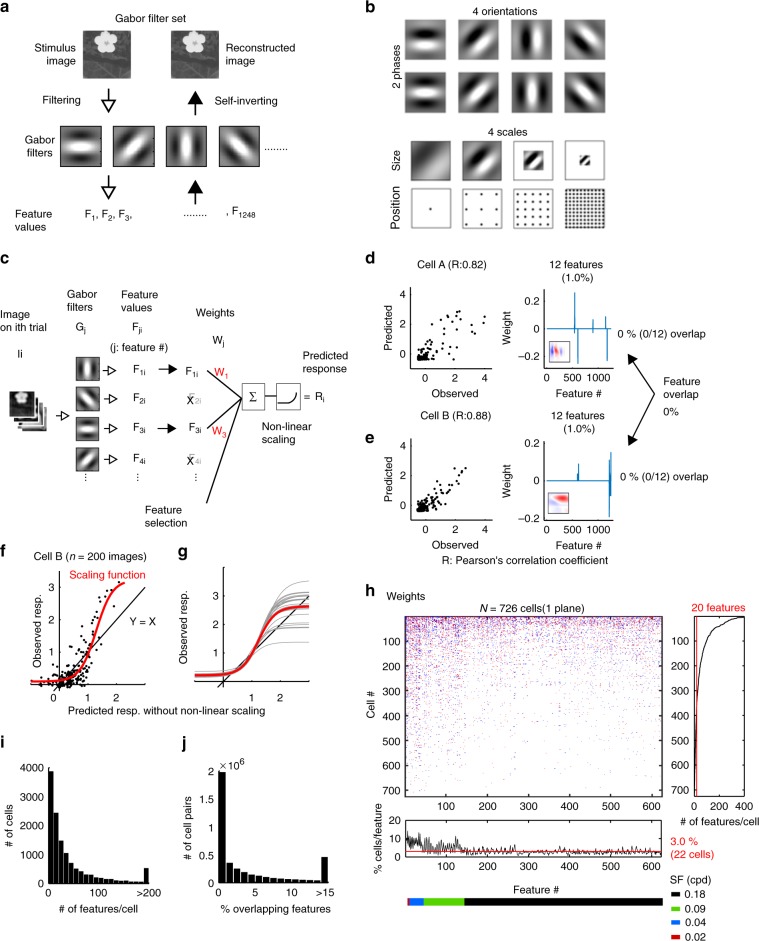
Small overlap in visual features among neurons. **a** Schematic of the transformation between a natural image and Gabor feature values. Each natural image was subjected to Gabor filters to obtain the feature values. Conversely, a set of feature values was transformed into an image. **b** Schematics of the Gabor filters. **c** Schematic of the encoding model. I*i*, stimulus image in the *i*th trial. G*j*: the *j*th Gabor filter. F_*ji*_: the *j*th feature value obtained from I*i*. W_*j*_, the weight for the *j*th Gabor feature; R_*i*_, the predicted visual response to I*i*. **d**, **e** Response prediction in two neurons. Left: comparison between the observed and predicted responses. Right: weight parameters. The weights of one of ten models (each model corresponds to one of the ten-fold CVs) are shown. Insets: Forward filters (weighted sums of Gabor filters; red: positive values; blue: negative values). **f** The observed responses against the responses predicted by the linear step without nonlinear (NL) scaling in the neuron shown in (**e**). Red: NL scaling function curve. **g** NL scaling curves across planes. Gray: the averaged NL scaling curve across cells in each plane. Red: the averaged curve across planes (*n* = 24 planes). **f**, **g** The black line indicates *y* = *x*. **h** Upper left: Raster plot of the weights in the plane illustrated in Fig. 1c (red: positive weights, blue: negative weights). The median values for the models of the 10-fold CVs are shown. Right: the number of features used for each cell. Bottom: the percentage of cells for which each feature was used in the response prediction. Colored bar: the SF of the features. Red lines: median values. Half of the Gabor features are shown for visibility, but the remaining features were included in the data shown in the right panel. **i** Distribution of the number of features in each cell (*n* = 12,755 cells). **j** Distribution of the percentages of features that overlapped between cells (*n* = 3,993,653 cell pairs). **d**–**f** Each dot indicates a response to one image. The stimulus images in **a** and **c** are adapted from the database in ref. ^56^ with permission. Source data are provided as a Source Data file.

We constructed an encoding model for the visual responses of an individual neuron to examine the visual features represented by each neuron. To extract the visual features from the natural images, we used a set of Gabor wavelet filters with self-inverting properties (1248 filters, Fig. 2a, b, see Methods section). Natural images (**I**) were subjected to Gabor filters (**G**_fwd_) and transformed into sets of Gabor feature values (**F**, see Methods section).

1 $${\mathbf{F}} = {\mathbf{G}}_{{\mathbf{fwd}}}\,{\mathbf{I}}$$

For each neuron, we first selected the Gabor features that exhibited strong correlations with the visual response. The correlation threshold for the selected feature was adjusted to maximize the visual response prediction in each neuron (Fig. 2c, Supplementary Fig. 2a, b). Then, the visual responses of a single cell (**R**^*k*^, the *k*th cell’s responses) were represented by a linear regression of the selected feature values (**F**_select_) followed by nonlinear scaling (NL), Fig. 2c, see Methods section).

2 $${\mathbf{R}}^k = {\mathrm{NL}}({\mathbf{W}}^k\,{\mathbf{F}}_{{\mathrm{select}}} + b^k)$$

Parameters of the regression model (weights, **W**^*k*^ and a bias, *b*^*k*^) were estimated with 90% of the dataset, and the prediction performance was estimated with the remaining 10% of the dataset (ten-fold cross-validation (CV)), where **W**^*k*^ and *b*^*k*^ were estimated in each CV. A model was obtained independently for each cell (i.e., for each *k*).

In example neurons (Fig. 2d, e), the response prediction performances (the correlation coefficients between the observed and the predicted responses) were 0.82 and 0.88. These neurons were represented by 12 (out of 1248) Gabor features (Fig. 2d, e, right panels). We defined a forward filter as a weighted sum of the Gabor filters; the forward filters of the example neurons were spatially localized (Fig. 2d, e, insets in the right panels).

The median prediction performance of the encoding model was 0.32 (0.15–0.50) in the example plane presented in Fig. 1 (median (25–75th percentiles); *n* = 726 cells) and 0.21 (0.06–0.42) in all cells across planes (*n* = 12,755 cells, Supplementary Fig. 2d). The nonlinear scaling suppressed weak predicted responses and enhanced strong predicted responses (Fig. 2f, g), suggesting that this nonlinear step enhanced the sparseness of the predicted response obtained from the linear step. The nonlinear step also slightly enhanced the performance (Supplementary Fig. 2c).

Individual neuron encoded a small number of Gabor features. On average, each neuron encoded 21 (8–51) features in all recorded neurons (*n* = 12,755 cells, Fig. 2h, i, Supplementary Fig. 2e). The Gabor features encoded by each neuron were spatially localized and had similar orientations (Supplementary Fig. 3a–d). The regression weights of the Gabor features in the encoding model were positively correlated with the similarity between the corresponding Gabor filter and the RF structure estimated using the regularized inverse method (see Methods section, Supplementary Fig. 3e–h)^32–34^.

The Gabor features only slightly overlapped between neurons (Fig. 2j). In the example neurons (Fig. 2d, e), none of the features overlapped. For all neuron pairs, the median overlap was 1.0% (0.0–5.6%) (relative to the features represented by each cell; Fig. 2j, Supplementary Fig. 2f). The feature overlap between neurons was positively correlated with the similarity of the forward filter (pixel-to-pixel correlation between forward filters; Supplementary Fig. 3i, j). Furthermore, the features encoded by neuronal population were diverse, which was reflected in the distribution of forward filter similarity between cells (correlation coefficient between forward filters: 0.0 (−0.03–0.04); Supplementary Fig. 3k). Thus, the Gabor features encoded by individual neurons were highly diverse and only slightly overlapped.

This analysis also revealed how the individual Gabor features were encoded across neurons (Fig. 2h). As the spatial frequency (SF) of the Gabor filter increased (i.e., the scale decreased), the corresponding feature contributed to the visual responses of fewer neurons (Fig. 2h bottom), reflecting that Gabor filters with a low SF covered more of the neuron’s RF, whereas Gabor filters with a high SF affected the responses of fewer neurons. Furthermore, almost all features contributed to the responses of at least one cell (Supplementary Fig. 2g).

### Image reconstruction from the population activity

**Fig. 3:**
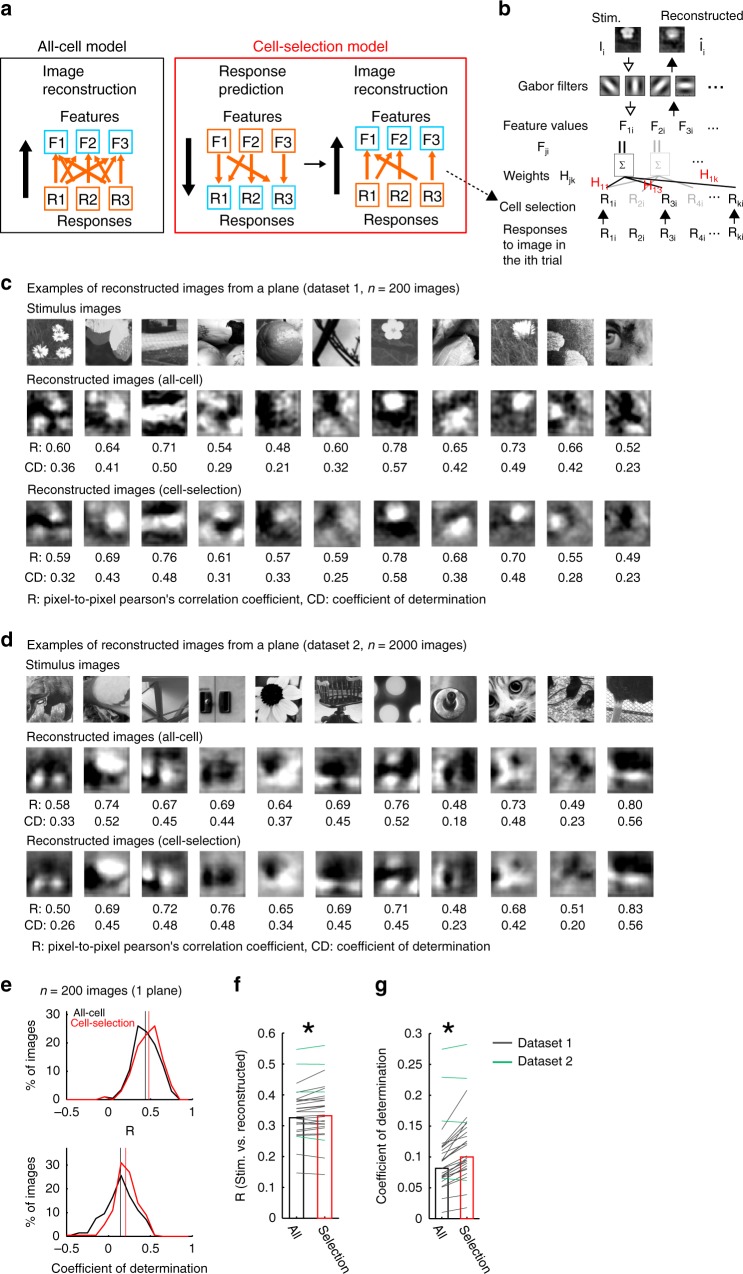
Image reconstruction from population activity. **a**, **b** Schematic of the image reconstruction models. **a** In the all-cell model, each feature value was reconstructed by all cells. In the cell-selection model, the feature value was reconstructed by selected cells for each feature. The cell selection was based on the response prediction model for each cell; each cell participates in the reconstruction of features that the cell encodes in the response prediction model. **b** Details of the image reconstruction model. For each Gabor feature, *j*, feature values (F_*ji*,_ *i:* trial number across stimuli and trials, *j*: Gabor feature number) were independently regressed (weights: H_*jk*_, *k*: cell number) by multiple cell responses (R_*ki*_) to the image (I*i*) in the *i*th trial. Then, a set of reconstructed features (F_1*i*_, F_2*i*_, …, F_1248*i*_) was transformed into an image $({\hat{\mathrm{I}}}_i)$. The flow of the reconstruction model is represented by black arrows from the bottom to the top. **c** Examples of reconstructed images from the main datasets (dataset 1; 200 images). Stimulus images presented during imaging (top), images that were reconstructed using the all-cell model (all cell, middle) and using the cell-selection model (cell selection, bottom) are shown. Each reconstructed image was averaged across trials. The reconstruction performances (R and CD) were computed for each trial, and trial-averaged performances are presented below each reconstructed image. **d** Examples of reconstructed images from other datasets (dataset 2; 1000–2000 images). **e** Distributions of R (top) and CD values (bottom) for the all-cell model (black lines) and the cell-selection model (red lines) in the example plane shown in Figs. 1 and 2 (*n* = 200 images reconstructed using 726 cells from a plane). Vertical lines indicate median values. **f**, **g** R **f** and CD **g** of dataset 1 (black lines and bars) and of dataset 2 (green lines) across planes. *p = 0.006 in **f** and *p* = 1.8 × 10^−5^ in **g** using the signed-rank test (*n* = 24 planes for dataset 1). Some of the stimulus images in **b**–**d** are adapted from the databases in refs. ^56–58^ with permission. Source data are provided as a Source Data file.

We next examined whether the features encoded in a neural population represent the visual contents of the natural images. To examine this, we reconstructed stimulus images from neuronal activity^15–19^. In the image reconstruction model, each Gabor feature value (**F** ^*j*^, the *j*th Gabor feature value) was subjected to a linear regression of the activity of multiple neurons (**R**) with model parameters of weights (**H** ^*j*^) and a bias term (*c* ^*j*^; Fig. 3a, b).

3 $${\mathbf{F}}^{\,j} = {\mathbf{H}}^j\,{\mathbf{R}} + c^{\,j}$$

Each Gabor feature value was independently reconstructed (i.e., **H** ^*j*^ and *c* ^*j*^ were estimated for each feature, *j*). Then, the sets of reconstructed feature values (${\hat{\mathbf{F}}} = [{\hat{\mathbf{F}}}^1; \ldots ;{\hat{\mathbf{F}}}^{1248}]$, ${\hat{\mathbf{F}}}^{\,j}$: reconstructed *j*th feature values) were transformed into images (**I**) based on almost self-inverting property of the Gabor filters (Gabor filter matrix for image reconstruction: **G**_rev_; Figs. 2a and 3b, see Methods section).

4 $${\hat{\mathbf{I}}} = {\mathbf{G}}_{{\mathrm{rev}}}{\hat{\mathbf{F}}}$$

The reconstruction performance was estimated with a different test dataset from the training dataset used in the regression analysis (ten-fold CV with the same data split as in the encoding model. **H** ^*j*^ and *c* ^*j*^ were estimated in each CV).

We first used a model in which each feature value was reconstructed from all neurons (all-cell model, Fig. 3a). In the example plane (*n* = 726 neurons, presented in Figs. 1 and 2), the rough structures of the stimulus images were reconstructed from the population activity (Fig. 3c). The pixel-to-pixel correlation between stimulus and reconstructed images (similarity of image patterns) was 0.43 (0.35–0.55) (median (25–75th percentiles) of 200 images (dataset 1) in the example plane (Fig. 3e upper panel) and 0.33 (0.30–0.37) across all planes (*n* = 24 planes, Fig. 3f). The coefficient of determination (CD, goodness of model prediction, see Methods section) was 0.14 (0.02–0.26) in the example plane and 0.08 (0.07–0.1) across planes (Fig. 3e, bottom, and 3g). We applied the same method for another dataset (dataset 2, 1000–2000 images that did not include original 200 images). The performances of dataset 2 were similar to those of the original dataset (R: 0.45 (0.34–0.52), CD: 0.19 (0.11–0.25), *n* = 4 planes from three mice (Fig. 3d, green lines in Fig. 3f, g). Thus, the visual contents of natural images were extracted linearly from the population activity.

We next used another reconstruction model, in which each feature was reconstructed from a subset of neurons that showed strong correlations between the feature values and responses (cell-selection model, Fig. 3a). In the cell-selection model, each neuron participated in the reconstructions of subsets of features that the neuron encoded in the response prediction model (Fig. 3b, see Methods section). The reconstruction performances of the cell-selection model were almost comparable or even slightly higher than those of the all-cell model (dataset 1, R: 0.33 (0.3–0.38), *p* = 0.006 by signed-rank test compared to all-cell model. CD: 0.10 (0.08–0.14), *p* = 1.8 × 10^−5^ by signed-rank test. Dataset 2, R: 0.45 (0.33–0.53), *p* = 0.9 by signed-rank test compared to all-cell model. CD: 0.19 (0.11–0.25), *p* = 0.9 by signed-rank test, Fig. 3c–g). We confirmed that the performances were not substantially affected by whether the test data were used for the cell-selection step or not (Supplementary Fig. 4, see Methods section).

In the cell-selection model, we changed the number of features for which each neuron participated in the reconstruction by manipulating the feature-selection threshold (Figs. 2c and 3a, Supplementary Fig. 5a) and examined how the reconstruction performance was affected by the number of features. The number of features of the original cell-selection model led to nearly optimal performances (Supplementary Fig. 5a–c). Thus, the cell-selection model captures nearly optimal feature-cell assignment for the reconstruction.

V1 neurons mainly represented low SF components of natural images. Reconstructions of lower SF components (0.02 and 0.04 cycle/degree, cpd) were better than those of higher spatial frequencies (0.09 and 0.18 cpd; R: 0.65, 0.53, 0.18, and 0.04, and CD: 0.24, 0.23, 0.02, and −0.03 for 0.02, 0.04, 0.09, and 0.18 cpd, respectively; Supplementary Fig. 5d–f). This may reflect the SF preferences of mouse V1 neurons^35^.

### A small number of neurons represents a natural image

We next examined whether a small number of responsive neurons mainly represented an image or whether the remaining neurons also contained information. For this purpose, we changed the number of neurons used in the reconstruction of each image and examined how the reconstruction performance was affected. In the image reconstruction from a subset of cells, the weights of the image reconstruction model were based on the weights of the cell-selection model. Because a different set of cells was used for each image, the weights were separately computed for each image (see Eq. (9) in Methods section).

**Fig. 4:**
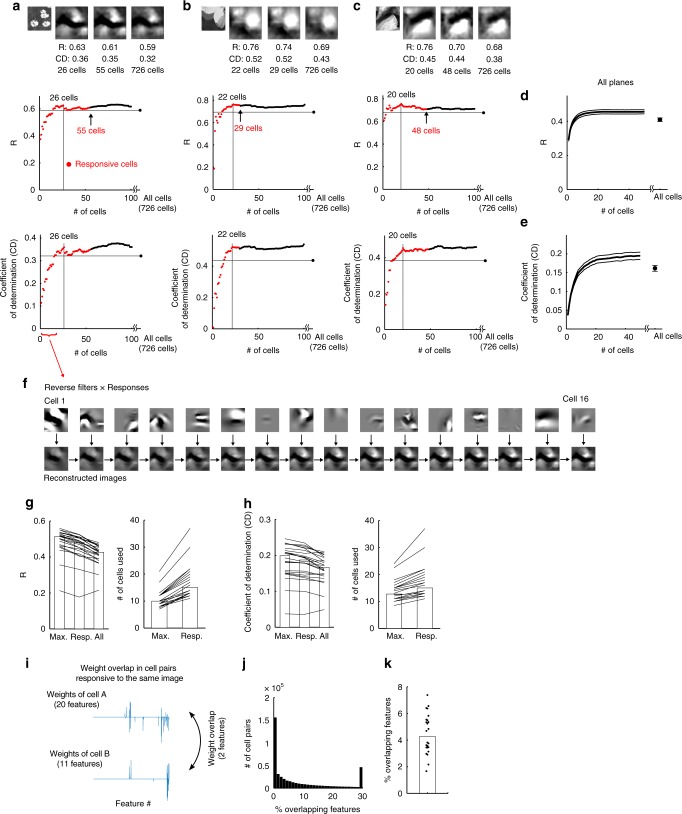
Image reconstruction by a small number of responsive neurons. **a**–**c** Top: examples of reconstructed images from a subset of responsive cells and from all cells. First panel: stimulus images. second–forth panels: reconstructed images from a subset of or all cells. Middle and bottom: reconstruction performances (middle, R; bottom, CD) plotted against the number of cells used for the reconstructions. The cells were first collected from the responsive cells (red dots) and then from the remaining cells (black dots). Horizontal lines: the performance from all cells. Vertical lines: the number of cells required for peak performance among all responsive cells. **d**, **e** Average performance curve (**d** R; **e** CD) plotted against the number of cells. Thick and thin lines indicate the means and the means ± S.E.M, respectively. **f** The contributions of the top 16 responsive cells to the image reconstruction shown in **a**. Top: reverse filters multiplied by the visual responses. Bottom: reconstructed images. **g**, **h** Left: median performances (**g** R; **h** CD) obtained from all cells (All), responsive cells (Resp.) and cells with peak performance (Max.). Right: the number of cells used for the reconstruction. (**g** left) *p* = 5.4 × 10^−5^ for Max. vs. Resp.; *p* = 1.2 × 10^−4^ for Resp. vs. All; *p* = 6.2 × 10^−5^ for Max. vs. All. (**g** right) *p* = 5.4 × 10^−5^. (**h** left) *p* = 5.4 × 10^−5^ for Max. vs. Resp.; *p* = 3.3 × 10^−4^ for Resp. vs. All; *p* = 1.3 × 10^−4^ for Max. vs. All. (**h** right) *p* = 1.7 × 10^−5^ using the signed-rank test. Each line indicates data for each plane, and bars indicate medians. Data for images that had at least ten responsive cells were used. **i**–**k** Weight overlap (i.e., features) between the cells that responded to the same image. **i** Schematic of the analysis. **j** Percentages of overlapping features for all-cell pairs responding to the same image. **k** The median percentages of overlapping features for all planes. Each dot indicates the median in each plane. **d**, **e**, **g**, **h**, **k** *N* = 24 planes. The stimulus images in **a**–**c** are adapted from the databases in ref. ^56^ and with permission. Source data are provided as a Source Data file.

A small number of responsive neurons primarily represented a natural image. In each example image, neurons were sorted by visual response amplitude (descending order) first among the responsive neurons (red dots in Fig. 4a–c) and then among the remaining neurons (black dots in Fig. 4a–c). The image was reconstructed by the top *N* neurons (*N* = 1–726 cells), and the reconstruction performances were plotted against the number of neurons used (Fig. 4a–e). On average, ~20 neurons reconstructed the images with a level of peak performance (Fig. 4d, e). The reconstruction performances of the responsive neurons were greater than those of all cells (left panels in Fig. 4g, h). Furthermore, among the responsive neurons for each image, the peak performances were obtained with highly responsive neurons that was fewer than the number of responsive neurons (right panels in Fig. 4g, h). Therefore, highly responsive neurons mainly represent the image, and the additional use of the remaining neurons even decreases the performance.

The features represented by individual neurons should be diverse in order to represent a natural image using a small number of neurons. Figure 4f illustrates how individual responsive neurons contribute to image reconstruction in the case presented in Fig. 4a. Each neuron had a specific pattern of contributions (reverse filter: sum of Gabor filters × weights), and the reverse filters varied across neurons (Fig. 4f, top panels) while partially overlapping in the visual field. For neuron pairs that were responsive to the same image, the number of overlapping Gabor features slightly increased compared to all pairs, but the percentage of overlapping features was still <5% (responsive cell pairs: 3.2% (0–13%) for all pairs and 4.3% (3.4–5.5%) across planes; all-cell pairs: 1.0% (0–5.6%) for all pairs and 1.0% (0.9–1.2%) across planes; Fig. 4i–k, cf. Fig. 2j, Supplementary Fig. 2f). These slightly overlapping and diverse features among neurons should be useful for image representation by a small number of neurons.

### Robust image representation by overlapping representation

**Fig. 5:**
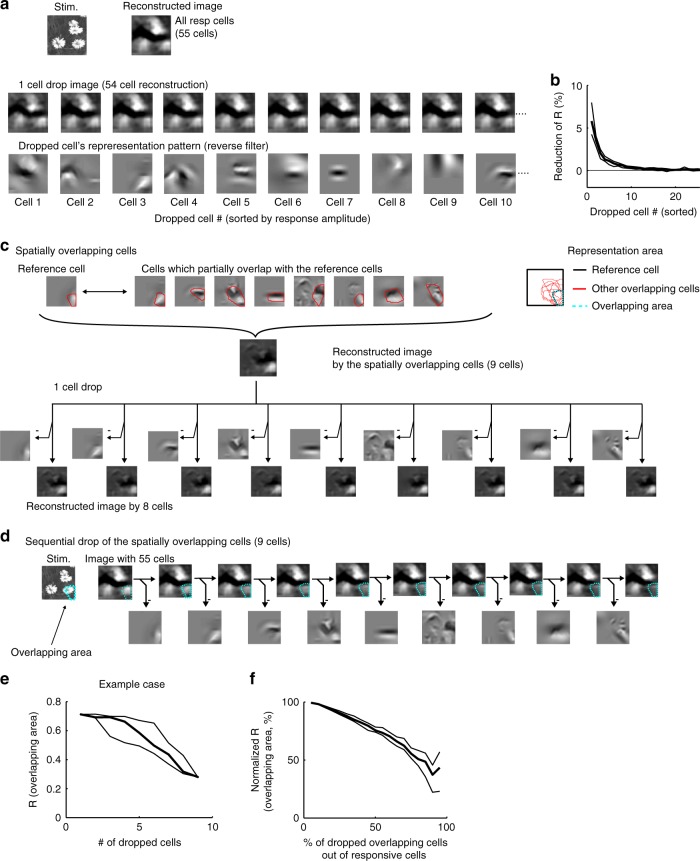
Robustness of image representation against cell drop. **a** Representative reconstructed images after dropping a single cell. Top panels: stimulus and reconstructed image obtained from all responsive neurons (55 cells). Middle panels: reconstructed images obtained after dropping a single cell. Bottom panels: representation patterns (reverse filters) of the dropped cells. The cell number (cell #) is the same as shown in Fig. 4f. **b** Reduction in reconstruction performance after removing a single cell. The cell #s on the *x*-axis are ordered from largest to smallest response amplitudes. The cell #s are in the same order as shown in Fig. 4d, e. Thick line: median. Thin lines: 25th and 75th percentiles; *N* = 24 planes. **c** Top panels: reverse filters of overlapping cells (nine example cells). The representation area of each neuron is contoured by the red line and overlaid on the right panel. Middle panel: reconstructed image obtained from the nine overlapping cells. Bottom panels: reconstructed images obtained from single cells (upper panels) and reconstructed images after dropping a single cell (lower panels). Dropping a single cell exerted only a small effect on the reconstructed images. **d** Representative reconstructed images obtained during the sequential dropping of the nine overlapping neurons. Cyan dotted lines indicate the overlapping area of the nine cells. The quality of the reconstructed image around the overlapping areas gradually degrades after each cell is dropped. **e**, **f** Plot of the R (or normalized R) for a local part of the reconstructed image (overlapping area) against the number (or percentage) of dropped cells for the representative case shown in **c**, **e** and for the summary of all data **f**. Data were collected and averaged across cells and across stimuli in each plane and then collected across planes. Thick lines: medians. Thin lines: 25th and 75th percentiles obtained across repetitions of random drops (*n* = 120 repetitions, **e**) or across planes (*n* = 24 planes, **f**). The stimulus images in **a** and **d** are adapted from the database in ref. ^56^ with permission. Source data are provided as a Source Data file.

We next examined whether a single image was robustly represented by a small number of responsive neurons. We computed the reconstruction performance after dropping individual responsive neurons (Fig. 5a, b; the cell # on the *x*-axis is the same as in Fig. 4d). Dropping a single cell reduced the reconstruction performance by ~5% for the best-responding neurons and did not change the performance for most neurons (middle panels in Fig. 5a, b). Thus, an image was robustly represented by responsive neurons against dropping a single cell.

This robustness against cell drop was due to the spatial overlap of reverse filters among responsive neurons (Fig. 5c). To analyze this, we selected a set of overlapping cells for each responsive neuron; the overlapping cells consisted of one responsive neuron as a reference (cell 1) and the responsive neurons whose representation areas partially overlapped with that of the reference cell (top panels in Fig. 5c and Supplementary Fig. 6a–d, see Methods section). The maximal reverse filter similarity between a responsive cell and other overlapping cells was 0.57 (0.41–0.70) (Supplementary Fig. 6d), indicating that each responsive neuron often had at least one neuron with a similar reverse filter. Then, we examined the effects of cell drop on the reconstruction of a local part of the image from the overlapping cells. Although dropping a single cell had almost no effect on the reconstructed local image (bottom panels in Fig. 5c), a sequential dropping of these cells gradually degraded the part of the image (Fig. 5d). The pixel-to-pixel correlation between the stimulus and the reconstructed image in the overlapping area gradually decreased as the number of dropped cells increased (Fig. 5e, f). Thus, the robust image representation was due to neurons with spatially overlapping representations.

### Reliable image representation across trials

We further analyzed whether the overlapping representation was useful in reducing the trial-to-trial variability in the image representation. Cortical neurons often show trial-to-trial response variability. However, the integration of activity among neurons with spatially overlapping or similar representations should reduce the variability in image representations across trials^36–38^.

**Fig. 6:**
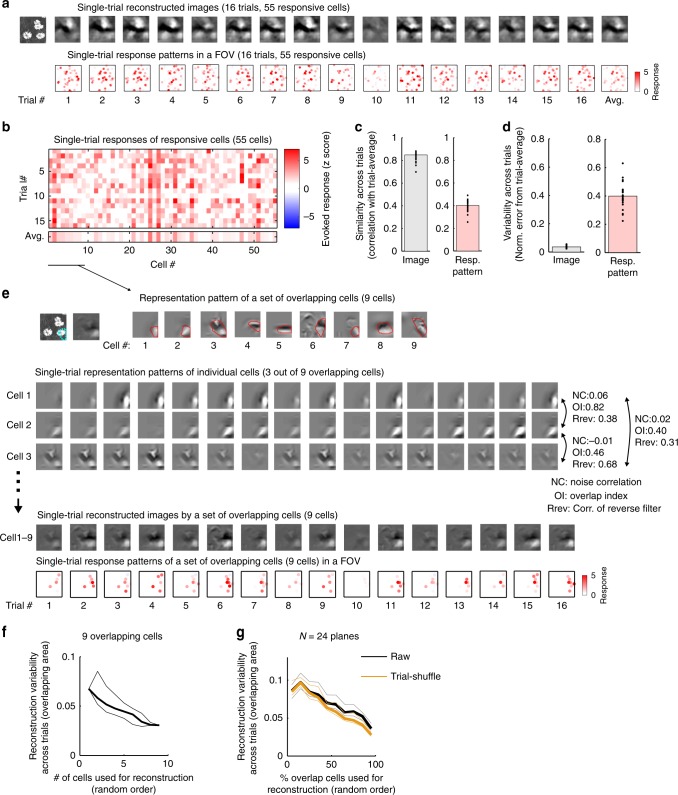
Reliable image representation across trials. **a** Examples of single-trial reconstructed images (top) and response patterns in a FOV (bottom). The first panel is a stimulus image, and the last panel is a trial-averaged image. FOV size: 507 micron each side. The color code for each dot indicates the response amplitude of each cell. **b** Single-trial evoked responses to the image in **a**. **c** Across-trial similarity of the reconstructed images (left) and the response patterns of responsive cells (right). The across-trial similarity was the Pearson’s correlation coefficient between a single-trial reconstructed image (or response pattern) and their trial average. **d** Across-trial variability of the reconstructed images (left) and the response patterns of responsive cells (right). The normalized squared error between a single-trial image (or response pattern) and their trial average was computed for the across-trial variability. **e** Reconstructed image from a set of overlapping cells (cell #1–9 in **b**). Upper left: stimulus image and trial-averaged reconstructed images from the nine overlapping cells. Cyan dotted line: the overlapping area. Upper right: representation (reverse filters) of the overlapping cells. Red line: the representation area. Cell #1 was the reference cell. Lower: single-trial representation patterns of three example cells (cell #1, 2, and 3) selected from the overlapping cells. Bottom: single-trial reconstructed images (upper) and single-trial response patterns in an FOV (lower) obtained from the nine overlapping cells. The brightness of each color dot in the lower panels indicates response amplitude of each cell. **f** Across-trial variability of a local part of reconstructed image (cyan dotted line in **e**) against the number of overlapping cells used for the reconstruction in the example case in **e**. Thick and thin lines are the median and 25th or 75th percentile, respectively (*n* = 200 random sequences of cell adding). **g** Across-trial variability against the percentage of overlapping cells used for the reconstruction. Black lines: raw data. Orange lines: trial-shuffled data. Thick and thin lines are median and 25th or 75th percentile, respectively. **c**, **d**, **g** *N* = 24 planes. The stimulus images in **a** and **e** are adapted from the database in ref. ^56^ with permission. Source data are provided as a Source Data file.

The pattern of the reconstructed image was reliably represented across trials regardless of the trial-to-trial response variability. In the example case (shown in Fig. 5), single-trial reconstructed images from all responsive neurons (55 cells) were generally stable across trials and blurred in only a few trials (e.g., trial 10, Fig. 6a, top). In contrast, the activity patterns of responsive neurons seemed variable across trials, although a few cells were reliably active (Fig. 6a, bottom, b). To evaluate the trial-to-trial reliability of the reconstructed images (or population responses), we used two measures, across-trial similarity and across-trial variability. Across-trial similarity was defined as the Pearson’s correlation between single-trial reconstructed images (or response patterns) and their trial average (Fig. 6c). Across-trial variability was the normalized squared error between single-trial reconstructed images (or response patterns) and their trial average (Fig. 6d, see Methods section). For all planes, the across-trial similarity of the reconstructed images was high (0.85 (0.81–0.89), Fig. 6c, left), whereas the across-trial similarity of the response was relatively low (0.40 (0.36–0.44), Fig. 6c, right). The across-trial variability of the image was relatively low (Fig. 6d, left), and that of the response pattern was relatively high (Fig. 6d, right).

We next examined how the images were reliably represented across trials. Neurons with partially overlapping or similar reverse filters will provide reliable representations across trials (for at least part of the image), if these neurons are active on different trials. In the example case, among the nine overlapping cells, the three example neurons (cell 1–3 in Fig. 6e) represented a partially similar pattern of the image (the correlations of reverse filters among the three neurons were 0.31–0.68), while they were active on different trials. Combining the nine overlapping cells reliably represent a local part of the image in most trials (Fig. 6e, bottom). In the example case, the across-trial variability of the local part of the reconstructed image gradually decreased as the number of cells used for the reconstruction increased (Fig. 6f). This was also observed in data from all planes (Fig. 6g, black lines), suggesting that multiple overlapping cells support reliable image representation.

We further examined the relationship between reverse filter overlap or similarity and noise correlations (see Methods section for the calculation, Supplementary Fig. 7a–c). Noise correlations were positively correlated with reverse filter correlations (correlation: 0.25, Supplementary Fig. 7d) and with signal correlations (correlation: 0.44, Supplementary Fig. 7e)^37,39^. However, the noise correlations were mainly distributed near 0 even in cell pairs with high overlap or reverse filter similarity (e.g., in pairs where the correlation of the reverse filter >0.5, the median noise correlation was 0.03; Supplementary Fig. 7b, c). Together, neurons with overlapping or similar reverse filters were almost independently active across trials, which was useful for reliable representation.

We also analyzed the effect of noise correlations on the reliable image representation. Removing the noise correlations (see Methods section) increased the reconstruction performances and decreased the across-trial variability (Supplementary Fig. 7g–i), suggesting that the noise correlations are rather detrimental to both reconstruction performance and the reliable image representation. In the relationship between the across-trial variability and the number of cells used for the reconstruction, the reduction in variability by removing noise correlations was much smaller than that by increasing the number of cells used for the reconstruction (Fig. 6g), suggesting that noise correlations have a relatively small impact on the reliable representation.

### Representation of multiple images in a population

**Fig. 7:**
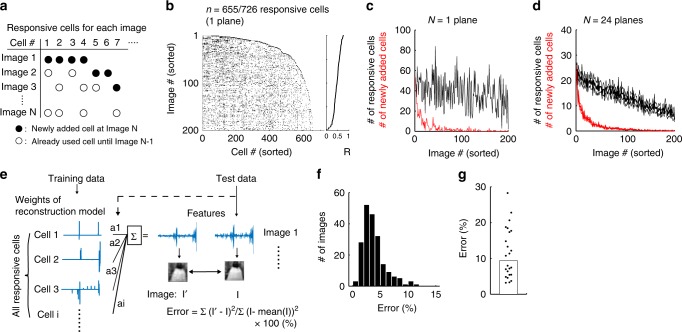
Representation of multiple images in a population. **a** Schematic of the analysis. Responsive neurons (open and closed circles) were plotted for each image. Closed circles: responsive cells plotted for the first time for image *N*. Open circles: responsive cells that had already been plotted for image 1 – (*N* − 1). *N*, image number. **b** Raster plots of responsive cells for each image in the representative plane shown in **a** (*n* = 655/726 responsive cells). The image #s are sorted by the reconstruction performance (right panel). For each line, cells that did not respond to the previously plotted images are added to the right side. As the image # increases, the number of newly added cells decreases, and the cell # quickly reaches a plateau, indicating that many images are represented by a combination of cells that responded to other images. **c**, **d** The number of responsive cells (black line) and of newly added responsive cells (red line) are plotted against the image # for the case shown in **b**, **c** and averaged across planes **d**. The number of newly added cells quickly decreases as the image # increases. Three lines in each color indicate the mean and the mean ± S.E.M. **e** Schematic of the analysis. The feature set of each natural image was linearly regressed by the weights of the reconstruction model (the cell-selection model) from all the responsive cells in each plane. The weights of the reconstruction model were obtained based on a training dataset, and the target image was selected from a test dataset. The fitting error (%) was computed for each image. If the features encoded in all responsive cells were sufficient to represent natural images, the weights of the responsive cells should work as basis functions to represent the visual features of the natural images. **f** Distributions of the errors for all images in the example plane (shown in other figures). **g** The median error (%) across planes. Each dot indicates the median of each plane. **d**, **g** *N* = 24 planes. The stimulus image in **e** is adapted from the database in ref. ^57^ with permission. Source data are provided as a Source Data file.

We next examined how multiple natural images were represented in a population of responsive neurons (Fig. 7a–d). In the example plane (*n* = 726 cells), natural images were sorted by reconstruction performance (*y*-axis in Fig. 7a, b), and the cells responding to each image are plotted in each row. First, as the number of images increased, new responsive cells were added, and the total number of responsive cells used for the reconstructions quickly increased (right end of the plot on each row, Fig. 7b). At ~50 images, the number of newly added responsive cells quickly decreased, and the increase in the total number of responsive cells slowed, indicating that the newly added image was represented by a combination of the already plotted responsive cells (i.e., neurons that responded to other images), due to the small overlap in responsive cells between images (Fig. 1j). In summary, the number of newly added cells quickly decreased to zero as the number of images increased (red lines in Fig. 7c, d). Therefore, although only ~5% of responsive neurons overlapped between images (Fig. 1j), this small overlap is useful for the representation of many natural images by a limited number of responsive neurons.

We also analyzed whether reverse filters encoded by the responsive neurons can represent any natural image as basis functions. If so, a set of features of the natural image will be accurately represented by a linear regression of the weights (i.e., feature sets) of responsive neurons in the cell-selection model independent of actual responses (see Methods section, Fig. 7e). Fitting errors were computed in the image space. The median error was <10% for all images and all planes (3.3% (2.3–4.6%) for the example plane and 9.3% (5.4–18%) for all planes; Fig. 7f, g). Thus, the Gabor features encoded by responsive neurons in a local population can accurately represent the visual contents of natural images.

### Image representation in awake mice

**Fig. 8:**
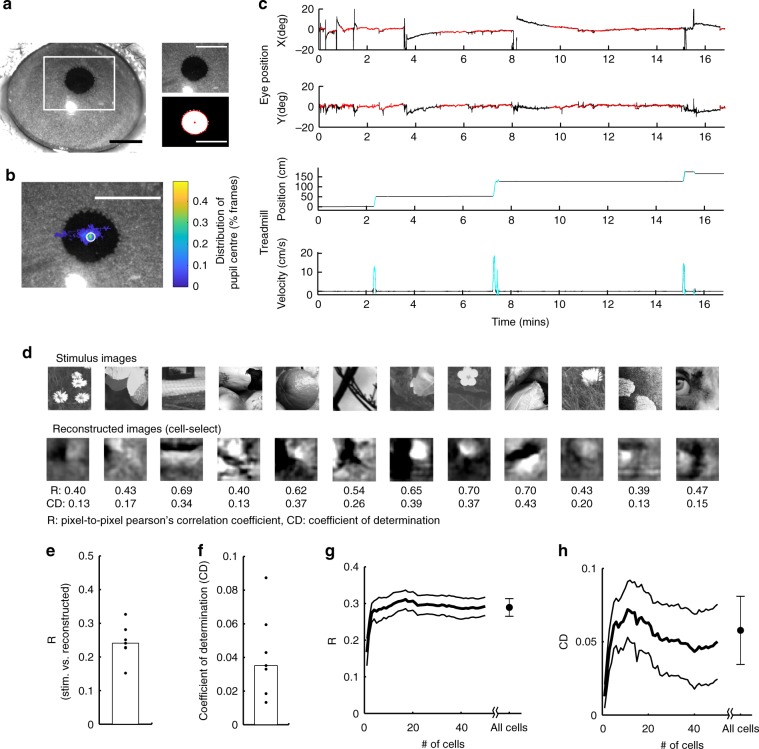
Image representation in awake mice. **a**, **b** Schematic of eye position analysis. **a** Image of a right eye. The white rectangle indicates the area recorded during imaging and analyzed offline (left). The recorded image (upper right panel) was binarized, and the pupil was fitted with an ellipse (red contour in lower right panel). The center of the ellipse was used to estimate the eye position (red dot). **b** Distribution map of eye position during imaging overlaid onto the image in **a**. The peak position of the distribution was detected, and the data were used for subsequent analyses only when the eye stayed around the peak position (white circle, <3.5 degrees or ~70 microns on the image). Scale bars: 1 mm. **c** Examples of eye position and locomotion state during imaging. Upper two panels: horizontal (*X*) and vertical (*Y*) eye positions. The red lines indicate time points at which the eye stayed around the peak position (inside the white circle in **b**). Lower two panels: position and velocity of a disc-type treadmill. The cyan lines indicate time points at which the mouse ran (velocity >2 cm/sec). **d**–**h** Image reconstruction by the cell-selection model in awake mice. **d** Examples of the reconstructed images. **e**, **f** Reconstruction performances (**e** pixel-to-pixel correlation, R. **f** coefficient of determination, CD); *N* = 7 planes. **g**, **h** R **g** and CD **h** versus the number of neurons. A single image was reconstructed by a small number of neurons. The thick black line and gray lines indicate the means and the means ± S.E.M, respectively (*n* = 6 planes). Only data for images that included at least five responsive cells were used. The stimulus images in **d** are adapted from the databases in refs. ^56,57^ with permission. Source data are provided as a Source Data file.

We finally examined whether the results obtained from anesthetized mice could be generalized to awake mice. We performed an additional experiment with awake, passively viewing mice that expressed GCaMP6s in the cortical cells (see Methods section)^40,41^. We recorded eye positions and locomotion states (running and resting) during imaging and used data only when the eye was still (<3.5 degrees, Fig. 8a–c, see Methods section).

The main results obtained from the anesthetized mice were also observed in the awake mice (Fig. 8d–h, Supplementary Figs. 8 and 9). Most cells were visually responsive for at least one image (82% (79–94%), *n* = 7 planes from three mice), whereas only a small percentage of cells were responsive for each image (1.5% (1.4–2.7%); Supplementary Fig. 8a–c). A single image was linearly decodable from a small number of responsive cells (Fig. 8d–h, Supplementary Fig. 8d–i). Furthermore, reconstructed images were robust to dropping a cell (Supplementary Fig. 8j, k) and reliable across trials (Supplementary Fig. 9). Therefore, the results in anesthetized mice could be generalized to those in awake, passively viewing mice.

We also separately analyzed the reconstruction performance from excitatory and inhibitory cells. In some experiments, we used the mice that expressed tdTomato in inhibitory cells (gad2-cre × Ai14)^42,43^ and identified excitatory and inhibitory cells based on the tdTomato (*n* = 5 planes from two mice). Excitatory cells were more visually responsive than inhibitory cells (Supplementary Fig. 10a–c). Furthermore, reconstruction performances by inhibitory cells were low compared to those only by excitatory cells and by all cells, whereas performances by excitatory cells were comparable to those by all cells (Supplementary Fig. 10d, e). Therefore, the images are mainly represented by excitatory cells at least under our experimental conditions.

We further examined how image reconstruction was affected by the locomotion state, which modulates V1 activity^44,45^. Although visual responses during running were greater than during resting (Supplementary Fig. 11a, b), the response patterns of a population of responsive cells were not largely different during running and resting (Supplementary Fig. 11c). Consequently, the reconstructed image patterns were not substantially affected by the locomotion state (Supplementary Fig. 11d, e).

## Discussion

Visual responses to natural images are sparse in V1 (refs. ^2,3,5–9,14,46^). We confirmed that a single natural image activated only a small percentage of neurons. According to the encoding model analysis, the visual responses of individual neurons were sparser than predicted from a linear model (Fig. 2f, g). Information has been proposed to be easily decoded from sparse representations^4^. Indeed, sparse activity increases the discriminability of two natural scenes by rendering the representations of the two scenes separable^5^. Our results extend this finding by showing that information about visual contents represented by sparsely active neurons are linearly accessible, suggesting that downstream areas easily and optimally decode images by collecting the activity of highly responsive neurons in the sparse representation.

The visual features encoded by individual neurons should be diverse, in order to ensure that a small number of active neurons represent the complex visual features of the image. Although the RF structures of mouse V1 have already been reported^21,22,33,34^, their diversity has not been analyzed with respect to natural image representation. In the present study, the visual features represented by sparsely active neurons were sufficiently diverse to represent the visual contents of natural images (Fig. 7e–g). Computational models for natural image representation suggest that sparse activity and the number of available neurons affect the diversity of RF structure^20,47–49^.

We also revealed how multiple natural images were represented in a local population. A single natural image activated specific subsets of neurons, whereas most neurons responded to at least one of the images, supporting the sparse, distributed code proposed in a previous study^10^. Thus, most V1 neurons are involved in visual processing. Due to the small overlap of responsive neurons between images (Fig. 1i), many natural images were represented by a limited number of responsive neurons (Fig. 7a–d). Furthermore, the RFs of all responsive neurons were sufficient to represent all the natural images used in the present study (Fig. 7e–g). Together, any natural image could be represented by a combination of responsive neurons. These findings also suggest that the representation of multiple natural images is high dimensional, consistent with a report about high-dimensional representation in mouse V1^50^. Thus, a single natural image can be low dimensionally represented in a high-dimensional representation space for a large number of natural scenes.

Sparsely active neurons reliably represented an image across trials, regardless of trial-to-trial response variability. Although a computational model proposed sparse and overcomplete representation as the optimal representation of natural images by unreliable neurons^20^, this model has never been investigated experimentally. The reliable representation was mainly achieved by diverse, partially overlapping representations, consistent with overcomplete representation. The RF subregions of some V1 neurons partially overlap^21^, which may be useful for reliable image representation. The reliable representation was also likely to be helped by almost independent activity across trials among neurons with similar RFs. Our results suggest a new representation scheme in which information is reliably represented, while the corresponding neuronal patterns change across trials. This model seems to be similar to “drop-out” in deep learning^51^ and may be useful for avoiding overfitting and local minimum problems in learning.

## Methods

### Preparation for two-photon imaging in anesthetized mice

All experimental procedures were approved by the local Animal Use and Care Committee of Kyushu University and the University of Tokyo. C57BL/6 mice (male and female) were used (Japan SLC Inc., Shizuoka, Japan). Mice were anesthetized with isoflurane (5% for induction, 1.5% for maintenance during surgery, ~0.5% during imaging with a sedation of <0.5 mg/kg chlorprothixene, Sigma-Aldrich, St. Louis, MO, USA). The skin was removed from the head, and the skull over the cortex was exposed. A custom-made metal plate for head fixation was attached with dental cement (Super Bond, Sun Medical, Shiga, Japan), and a craniotomy (~3 mm in diameter) was performed over left V1 (center position: 0–1 mm anterior to lambda, +2.5–3 mm lateral to the midline). A mixture of 0.8 mM Oregon Green BAPTA1-AM (OGB1, Life Technologies, Grand Island, NY, USA) dissolved in 10% Pluronic (Life Technologies) and 0.025 mM sulforhodamine 101 (ref. ^52^; SR101, Sigma-Aldrich) was pressure-injected with a Picospritzer III (Parker Hannifin, Cleveland, OH, USA) at a depth of 300–500 µm from the brain surface. The cranial window was sealed with a coverslip and dental cement. The imaging experiment began at least 1 h after the OGB1 injection.

### Preparation for two-photon imaging in awake mice

In awake mouse experiments, we used two lines of transgenic mice: Thy1-GCaMP6s (GP4.3) transgenic mouse^41^ (JAX #024275, *n* = 1 mouse, two imaging planes) and the mice obtained by crossing gad2-ires-cre mice^43^ (JAX #010802) with Ai14 mice^42^ (JAX #007914; Gad2-Ai14, *n* = 2 mice, five imaging planes). Thy1-GCaMP6s mice express GCaMP6s^40^ in cortical neurons. Gad2-Ai14 mice express tdTomato in almost all inhibitory neurons^43^.

GCaMP6s was introduced into Gad2-Ai14 mice via an adeno-associated virus (AAV). The Gad2-Ai14 mice were anesthetized with isoflurane as described above. A small incision was made on a sculp, and a small hole (<0.3 mm diameter) was made in the skull over left V1. AAV2/1-syn-GCaMP6s^40^ (vector core; University of Pennsylvania, Philadelphia, PA, USA) was injected into V1 through the hole (titer: 3.0–5.0 × 10^12^ genomes/ml, volume: 500 nl, depth: 250-300 micron from a brain surface). After suturing the incision, the Gad2-Ai14 mice were recovered at least 3 days after the injection. The mice were anesthetized with isoflurane to attach a metal plate for head fixation and to make a cranial window as described above. The mice were recovered after the surgery.

The mice were daily habituated with a head fixation on a disc-type treadmill. Duration of the head fixation started with a few minutes and gradually prolonged up to ~2 h over several days. If mice were calmly head-fixed for 2 h without any stressful sign, imaging experiments started on the next day. The imaging started at least 1 week after the cranial window surgery, and 3 weeks after the virus injection for the gad2-Ai14 mice.

### Two-photon Ca^2+^ imaging

Imaging was performed with a two-photon microscope (A1R MP, Nikon, Tokyo, Japan) equipped with a 25× objective (NA 1.10, PlanApo, Nikon) and Ti:sapphire mode-locked laser (MaiTai Deep See, Spectra Physics, Santa Clara, CA, USA)^53,54^. OGB1, SR101, GCaMP6s, and tdTomato were excited at a wavelength of 920 nm. Emission filters with a passband of 525/50 nm were used for the OGB1 and GCaMP6s signals, and filters with a passband of 629/56 nm for the SR101 and tdTomato signals. The fields of view (FOVs) were 338 × 338 µm (10 planes from seven anesthetized mice) and 507 × 507 µm (14 planes from seven anesthetized mice and seven planes from 3 awake mice) at 512 × 512 pixels. The sampling frame rate was 30 Hz using a resonant scanner.

### Monitoring of eye and treadmill motion in awake mice

In awake mice experiments, right eye images and rotation of the disc-type treadmill were recorded during the imaging. The right eye was monitored with a USB camera (NET New Electronic Technology GmbH, Germany). The treadmill rotation was monitored with a rotary encoder (OMRON, Japan). The eye images, the encoder signals and time stamps of frame acquisition of the two-photon imaging were simultaneously recorded using a custom-written program in LabView (National Instruments, Austin, TX, USA).

### Visual stimulation

Before beginning the recording session, the retinotopic position of the recorded FOV was determined using moving grating patches (lateral or upper directions, 99.9% contrast, 0.04 cycle/degrees, 2 Hz temporal frequency, 20 and 50 degrees in diameter), while monitoring the changes in signals over the entire FOV. The lateral or upper motion directions of the grating were used to activate many cells because the preferred directions of mouse V1 neurons are slightly biased toward the cardinal directions^54,55^. First, the grating patch of 50 degrees in diameter was presented in 1 of 15 (5 × 3) positions that covered the entire monitor to roughly determine the retinotopic position. Then, the patch of 20 degrees in diameter was presented on the 16 (4 × 4) positions covering an 80 × 80-degree space to finely identify the retinotopic position. The stimulus position that induced the maximum visual response of the entire FOV was set as the center of the retinotopic position of the FOV.

A set of circular patches of greyscale, contrast-enhanced natural images (200 image types in main datasets, dataset 1) was used as the visual stimuli for predicting the response and reconstructing the natural image (256 intensity level, 60 degrees in diameter, 512 × 512 pixels, with a circular edge (5 degrees) that was gradually mixed to a gray background). Contrasts of natural images were enhanced (>90%). Original natural images were obtained from the van Hateren Natural Image Dataset (http://pirsquared.org/research/#van-hateren-database)^56^ and the McGill Calibrated Color Image Database (http://tabby.vision.mcgill.ca/html/welcome.html)^57^. Square image patches (512 × 512 pixels) were obtained from around centers of the original images. Some original images were downsampled before the extraction of the center parts. We selected 200 images that had spatial structure for the final stimulus set and did not include images that had less spatial structure (e.g., almost flat image) and very high SF components throughout the image (e.g., fine texture) by visual inspection. The pixel-to-pixel correlation between images was 0.003 (−0.12–0.11) (median (25th–75th percentile), *n* = 200 images).

During image presentation, one image type was consecutively flashed three times (three 200 ms presentations interleaved with 200 ms of a gray screen), and the presentation of the next image was initiated after the presentation of the gray screen for 200 ms. Images were presented in a pseudorandom sequence, in which each image was presented once every 200 image types. Each image was presented at least 12 times (i.e., 12 trials) for anesthetized and ~40 times for awake mice in the entire recording session for one plane. We did not set a long interval between image flashes to reduce the total recording time and maximize the number of repetitions. In this design, the tail of the Ca^2+^ response to one image invaded the time window of the next image presentation (Fig. 1b). Although this overlap may have affected the visual responses to two adjacent images, the use of many trial repetitions with the pseudorandom image sequences and the sparse responses to natural images (Fig. 1) minimized the effects of response contamination between two consecutive images.

In another set of experiments with anesthetized mice, we used different image sets (1000–2000 images that did not contain the 200 images described above, *n* = 4 planes from three mice). The original images for these sets were derived from the image datasets described above, the Caltech 101 dataset (http://www.vision.caltech.edu/Image_Datasets/Caltech101/)^58^, a free image website (https://www.pakutaso.com/), and images that we photographed. In experiments using these image sets, each image was presented three to eight times.

Moving square gratings (eight directions, 0.04 cycles/degree, 2 Hz temporal frequency, 60-degree patch diameter) were presented at the same position as the natural image on the screen. Each direction was presented for 4 s interleaved by 4 s of the gray screen. The sequence of directions was pseudo-randomized, and each direction was presented ten times for anesthetized mice.

All stimuli were presented with PsychoPy^59^ on a 32-inch, gamma-corrected liquid crystal display monitor (Samsung, Hwaseong, South Korea) with a 60-Hz refresh rate, and the timing of the stimulus presentation was synchronized with the timing of image acquisition using a transistor–transistor logic pulse counter (USB-6501, National Instruments).

The entire recording session for one plane was divided into several sub-sessions (4–6 trials/sub-session and 15–25 min for each sub-session). Each sub-session was interleaved by ~5–10 min of rest time, during which the slight drift of the FOV was manually corrected. In anesthetized mice, the retinotopic position of the FOV was confirmed with the grating patch stimuli during the rest time every two or three sub-sessions, and the recording was terminated if the retinotopic position had shifted (probably due to eye movement). The recordings were performed in one to three planes of different depths and/or positions in each anesthetized mouse (1.7 ± 0.8 planes, mean ± standard deviation). In awake mice, the recording continued independent of the eye position and terminated if the mouse showed any stressful sign. The recording was performed in one plane per day, and one or two planes were obtained from each awake mouse.

For the analyses described below, the natural images were scaled such that the maximum (255) and minimum (0) intensities were 1 and −1, respectively, and the gray intensity (127) was 0. A square (43 × 43 degrees) positioned in the center of the natural image patch was extracted and downsampled to a 32 × 32-pixel image. The downsampled image was used to analyze the Gabor features, response predictions and image reconstructions.

### Analysis of eye positions and treadmill rotations

Each eye image for the awake mice was binarized based on pixel intensities. The contour of the binarized pupil area was fitted with an ellipse whose center was used as the eye position (Fig. 8a, b). The eye positions on the image were transformed to angular positions. In this transformation, a previously reported value was used for the radius of the mouse eye^60^. In the distribution of the eye position during the entire recording session, a peak position was manually selected. Only the time points at which the eye was within 3.5 degrees (or ~70 microns on the image) of the peak position were used for all analyses described below (except for the time course extraction of the Ca^2+^ signal from the two-photon imaging data).

In the analysis of the treadmill rotation, position signals from the rotary encoder on the treadmill were transformed to velocity and smoothed with a Savitzky–Golay filter. Running periods were defined as periods during which the velocity was >2 cm/sec.

### Analysis of two-photon imaging data

All data analysis procedures were performed using MATLAB (Mathworks, Natick, MA, USA). The recorded images were phase-corrected and aligned between frames. The average image across frames was used to determine the region of interests (ROIs) of individual cells. After removing the slow SF component (obtained with a Gaussian filter with a sigma of approximately five times the soma diameter), the frame-averaged image was subjected to a template matching method in which the 2D, difference of Gaussians (sigma1: 0.26 × soma diameter that was adjusted for zero-crossing at the soma radius, sigma2: soma diameter) was used as a template for the cell body. Highly correlated areas between the frame-averaged image and the template were detected as ROIs for individual cells. ROIs were manually corrected via a visual inspection. SR101-positive cells (putative astrocytes^52^) were removed from the ROI in data of anesthetized mice. For data of awake mice, a cross-correlation image^21^ and a max-projection image across frames were also used for the ROI detection.

The time course of the calcium signal in each cell was computed as an average of all pixels within an ROI. Signal contamination from an out-of-focus plane was removed using a previously reported method^54,61^. Briefly, a signal from a ring-shaped area surrounding each ROI was multiplied by a factor (contamination ratio) and subtracted from the signal of each cell. In anesthetized mice, the contamination ratio was determined to minimize the difference between the signals from a blood vessel and the surrounding ring-shaped region multiplied by the contamination ratio. The contamination ratios were computed for several blood vessels in the FOV, and the mean value for several blood vessels was used for all cells in the FOV. In awake mice, the contamination ratio was set to 0.7 for all cells following a previous study^40^, because it was difficult to identify the blood vessels in the GCaMP imaging.

After removing the out-of-focus signal, slow temporal frequency components (>60 sec/cycle) were removed from the time course of each cell (a Gaussian low-cut filter applied on the frequency domain for anesthetized data or a median low-cut filter for awake data), followed by smoothing with the Savitzky–Golay filter (forth order, 15 frame points length (~500 ms)). Then, the filtered time course (F_filtered_) was transformed to ratio change (*dF*/*F*) by using the 20th percentile value across frames (*F*) (*dF*/*F* = (*F*_filtered_ − *F*)/*F*). Frame-averaged activity (*dF*/*F*) during 200 ms baseline (six frames immediately before the stimulus) and during stimulus (average of the last 200 ms for each stimulus period) were used for subsequent analyses. The evoked response was obtained by subtracting the activity during baseline from that during stimulus. In anesthetized mice, data for moving grating from one plane was discarded because of a large drift of FOV during recording. In awake mice, only data for images that contained at least six trials were used for subsequent analyses.

### Analysis of visual responses

Visually responsive neurons were determined by one-way ANOVA (*p* < 0.01) with a dataset of *N* stimuli and one baseline (mean across stimuli) activity in each trial (size: *N* + 1 activity × no. of trials, *N*: the number of stimuli used for the analysis). To validate this criterion, ANOVA was applied to a randomized dataset in which data labels were shuffled in each trial. The false positive rate was only a small fraction of the percentage of the responsive cells (anesthetized: 85% responsive cells and 1.0% false positive rate. Awake: 82% responsive cells and 1.8% false positive rate).

For each responsive neuron identified by ANOVA, responsiveness for each image was determined by using a *t*-test (*p* < 0.01, comparison of activity between stimulus and baseline) and a trial-averaged evoked response (>10%). The evoked response threshold was used to reduce the false positive rate (Supplementary Fig. 1). The false positive rate was determined with the label-shuffled data. Without the evoked response threshold, the false positive rate was relatively high compared to the percentage of responsive cells per image (anesthetized: 3.2% for observed and 0.4% for shuffled data. Awake: 1.8% for observed and 0.3% for shuffled data). With the 10% amplitude threshold, the false positive rate decreased (anesthetized: 2.5% for observed and 0.1% for shuffled data. Awake: 1.5% for observed and 0.1% for shuffled data, Supplementary Fig. 1). Thus, we used the 10% evoked response threshold. For the dataset with 1000–2000 stimulus images (dataset 2), responsive cells were not determined because of fewer trials.

The population sparseness (s) was computed using the equation described in previous studies^2,3,62^ as follows: ${\mathrm{s}} = [1 - ({\sum} {{\mathrm{R}}i} )^2/({\mathrm{N}}_{{\mathrm{cell}}}{\sum} {{\mathrm{R}}i^2} )]/(1 - 1/{\mathrm{N}}_{{\mathrm{cell}}})$, where R*i* is the evoked response of the *i*th cell, and N_cell_ is the number of cells (*i* = 1, …, N_cell_). *Z*-scored evoked responses were used in the following analyses, including response prediction and image reconstruction (*z*-score was computed with responses across stimuli and trials in each cell).

### Gabor features

A set of spatially overlapping Gabor filter wavelets (*n* = 1248 filters) with an almost self-inverting feature was prepared to extract the visual features of the natural images^10,63,64^. The downsampled images were first subjected to the set of Gabor filters to obtain Gabor feature values. A single feature value corresponds to a single wavelet filter.

Gabor filters have four orientations (0, 45, 90, and 135 degrees), two phases, and four sizes (8 × 8, 16 × 16, 32 × 32, and 64 × 64 pixels) located on 11 × 11, 5 × 5, 3 × 3, and 1 × 1 grids (Fig. 2a, b). Therefore, the three smaller scale filters spatially overlapped with each other. The spatial frequencies of the four scale sizes of the Gabor wavelets were 0.02, 0.04, 0.09, and 0.18 cycle/degrees (cpd).

The Gabor filter set was almost self-inverting^63^, i.e., the feature values obtained by applying an image to the wavelet set could be transformed to the image by summing the filters after multiplying by the feature values.

5 $${\mathbf{F}} = {\mathbf{G}}_{{\mathbf{fwd}}}\,{\mathbf{I}}$$

6 $${\mathbf{I}}^\prime = {\mathbf{G}}_{{\mathbf{rev}}}\,{\mathbf{F}}$$

In Eq. (5) (corresponding to Eq. (1) in the main text), **F** is the feature value matrix (matrix size: *f* × *s*; *f*: the number of features, 1248, and *s*: the number of images), **G**_fwd_ is the Gabor filter matrix that transforms images to feature values, in which each row contains the reshaped 2D-Gabor filters (*f* × *p*; *p*: the number of the image pixels, 1024), and **I** is the downsampled stimulus image matrix (*p* × *s*). In Eq. (6) (corresponding to Eq. (4)), **I**′ is the reconstructed image matrix (*p* × *s*) from **F**, and **G**_rev_ is the Gabor filter matrix that transforms the features to images (*p* × *f*). In an ideal situation, ${\mathbf{G}}_{{\mathrm{fwd}}}^{ - 1}$ equals ${\mathbf{G}}_{{\mathrm{fwd}}}^{\mathrm{T}}$ (i.e., **G**_rev_ = ${\mathbf{G}}_{{\mathrm{fwd}}}^{\mathrm{T}}$. **A**^−1^ and **A**^T^ are inverse and transposed matrices of **A**, respectively.), resulting in **I**′ equaling **I** (ref. ^63^). However, our Gabor transformation was not perfect; the pixel-to-pixel correlation between **I** and **I**′ was 0.93 ± 0.026 (mean ± standard deviation, *n* = 200 images). To minimize the effect of this information loss on the evaluations of image reconstruction performance (see below), we used **I**′ instead of **I** as the target images for the evaluation of image reconstruction. The **G**_rev_ in Eq. (6) was different from ${\mathbf{G}}_{{\mathrm{fwd}}}^{\mathrm{T}}$ in Eq. (5) in terms of scaling; **G**_rev_ = *α* ${\mathbf{G}}_{{\mathrm{fwd}}}^{\mathrm{T}}$ (*α*: scaling factor. *α* was computed to minimize the sum of the mean squared error between **I**′ and **I**). The Gabor filters and the transformations were based on an open source program (originally written by Dr. Daisuke Kato and Dr. Izumi Ohzawa, Osaka University, Japan, https://visiome.neuroinf.jp/modules/xoonips/detail.php?item_id=6894).

### Encoding model (response prediction model)

In the encoding model, single-cell responses (**R**^*k*^ = [R_*ki*_], *k*: cell number and *i*: trial number across stimuli and trials. Size: 1 × N_trial_; N_trial_: the number of trials across stimuli and trials) were predicted using linear regression analysis of the selected Gabor feature values (**F**_select_ = [F_*ji*_], *j*: selected Gabor feature number. Size: *f*_select_ × N_trial_; *f*_select_: number of selected features, Fig. 2a–c, Supplementary Fig. 2a, b).

7 $${\mathbf{R}}^k = {\mathrm{NL}}({\mathbf{W}}^k\,{\mathbf{F}}_{{\mathrm{select}}} + b^k)$$

where **W**^*k*^ (=[W_*kj*_]; size: 1 × *f*_select_) is the weight vector, **F**_select_ (*f*_select_ × N_trial_) is the matrix of selected feature values, *b*^*k*^ (size: 1 × 1) is bias, and NL() is the nonlinear scaling function (Eq. (7) corresponds to Eq. (2)). The encoding model was created independently for each cell. The features used in the regression were determined as follows. First, Pearson’s correlation coefficients between the response and feature values were computed for each feature. Then, using one of the preset values for the correlation coefficient as a threshold (13 points ranging from 0.05 to 0.35, Supplementary Fig. 2a, b), only the more strongly correlated features were selected (feature selection) and used in the regression analysis. **W**^*k*^ and *b*^*k*^ were estimated to minimize the loss function: ${\sum} {\left( {{\mathbf{R}}^k - \left( {{\mathbf{W}}^k{\mathbf{F}}_{{\mathrm{select}}} + b^k} \right)} \right)} ^2 + \lambda {\sum} {{\mathbf{W}}^{k^2}}$ (*λ*: regularization parameter). This was solved by using Bayesian linear regression with an expectation-maximization algorithm that is approximately equivalent to linear regression with L2 regularization^65^. After the regression analysis, the nonlinearity of the predicted response was adjusted with a rectification step using the following function^34^: NL(*x*) = *A*/[1 + exp(*Bx* + *C*)] + *D*, where *A*, *B*, *C*, and *D* are parameters estimated using a built-in Matalb function (*lsqnonlin*). This step merely scaled the regression output without changing the regression parameters (**W**^*k*^ and *b*^*k*^).

The response prediction performance of the model was estimated by ten-fold CVs, in which the response data for 90% images were used to estimate the parameters, and the remaining data for 10% images were used to evaluate the prediction, (thus, **W**^*k*^ and *b*^*k*^ were estimated and fixed in each CV). In the ten-fold CVs, all images were used once as test data. The prediction performances were estimated using Pearson’s correlation coefficients between the observed (trial average) and predicted responses. Encoding models were created for all preset threshold values for feature selection, and the model that exhibited the best prediction performance was selected as the final model.

In the analysis of overlapping weights (i.e., feature) between two cells, the percentage of overlapping weights relative to the number of non-zero weights was computed for each cell and averaged between the two cells in the pair.

Using the same dataset as used in the encoding model, the RF structure was estimated for each cell using a regularized inverse method^32–34^ that employs one hyper parameter (regularized parameter). In the ten-fold CVs, the RF structure was estimated with the training dataset using one of the preset regularized parameters (13 logarithmically spaced points between 10^−3^ and 10^3^). The visual response was predicted using the estimated RF and test dataset. The prediction performance of visual response was estimated by determining Pearson’s correlation coefficients between the observed and the predicted responses. RFs were estimated for all values of the preset regularized parameters, and the value that resulted in the best predicted response was selected for the final RF model.

### Image reconstruction

For image reconstruction, the feature values obtained from each Gabor filter were linearly regressed by the single-trial activity of multiple cells. For each Gabor feature,

8 $${\mathbf{F}}^j = {\mathbf{H}}^j{\mathbf{R}} + c^j$$

where **F** ^*j*^ (= [F_*ji*_]) is the feature value from the *j*th Gabor filter (*j*: Gabor feature number, *i*: trial number across stimuli and trials. Size: 1 × N_trial_; N_trial_: the number of trials across stimuli and trials), **H** ^*j*^ (=[H_*jk*_]. Size: 1 × N_cell._ N_cell_: the number of cells) represents the weights, and **R** (=[R_*ki*_]. *k*: cell number. Size: N_cell_ × N_trial_)_._ is the response matrix. In the ten-fold CVs, the weights, **H** ^*j*^, and a bias, *c* ^*j*^, were estimated to minimize the loss function: $\sum ({\mathbf{F}}^{\,j} - ({\mathbf{H}}^j\,{\mathbf{R}} + c^{\,j}))^2 + \lambda \sum {\mathbf{H}}^{\,j^2}$, which was solved by using Bayesian linear regression with an expectation-maximization algorithm with the training dataset. Then, each Gabor feature value $({\hat{\mathbf{F}}}^{\,j})$ was reconstructed from the visual responses in the test dataset (ten-fold CV with the same data split as that in the encoding model. **H** ^*j*^ and *c* ^*j*^ were estimated and fixed in each CV). After each Gabor feature was independently reconstructed, sets of reconstructed feature values (${\hat{\mathbf{F}}} = [{\hat{\mathbf{F}}}^1; \ldots ;{\hat{\mathbf{F}}}^{1248}]$. Size: *f* × N_trial_) were transformed into images using Eq. (6).

In the all-cell model, each feature was reconstructed using all cells (Fig. 3a, left panel). In the cell-selection model, each feature was reconstructed using a subset of cells. For each feature reconstruction, cells were selected using the encoding model; if a cell was represented by *j*th feature in the encoding model (i.e., non-zero weight in jth feature in the Eq. (7)), the cell was selected for the *j*th feature reconstruction (Fig. 3a, right). In other words, each cell participated in the reconstruction of features that the cell encoded. When none of the cells were selected for feature reconstruction, the feature value was set to 0.

Reconstruction performance was evaluated using pixel-to-pixel Pearson’s correlation coefficients (R) and coefficients of determination (CD) between the stimulus and reconstructed images. CD was computed using the following equation: ${\mathrm{CD}} = 1 - \sum ({\mathbf{I}}^\prime - {\hat{\mathbf{I}}})^2/\sum ({\mathbf{I}}^\prime - {\mathbf{I}}_{{\mathrm{mean}}}^\prime )^2$ (${\hat{\mathbf{I}}}$: image reconstructed by the model, **I**′: stimulus image obtained by the transformation and reconstruction of the Gabor filters (Eq. (6)), and ${\mathbf{I}}_{{\mathrm{mean}}}^\prime$: mean pixel intensity of **I**′). R indicates the similarity of the image patterns between ${\hat{\mathbf{I}}}$ and **I**′, and CD indicates the goodness of model prediction reflecting differences in pixel intensities between ${\hat{\mathbf{I}}}$ and **I**′.

The cell-selection described above (i.e., feature selection in the encoding model) should overestimate the reconstruction performance because the test dataset was used for both the cell-selection and the performance evaluation of the reconstruction model. To precisely evaluate the performance of the cell-selection model, we used nested CV for the cell selection; a dataset was separated into 10% test, 9% validation, and 81% training sets, and the cell selection was performed with the validation and training sets. Then, the performance of the reconstruction model that was trained with both the validation and training sets was evaluated using the test dataset. The performance of the reconstruction model with nested CV was similar to that of the model without nested CV (Supplementary Fig. 4).

In the analysis of the overlapping weights (i.e., feature) between cells, the percentage of overlapping weights relative to the number of non-zero weights was computed for each cell and averaged between the two cells in the pair.

We independently obtained the weights of the image reconstruction model (**H** = [**H**^1^;…; **H**^1248^], size: *f* × N_cell_), the weights of encoding model (**W** = [**W**^1^; …; **W**^Ncell^]^**T**^, size: *f* × N_cell_) and RF by the pseudoinverse method. We chose the scheme of the image reconstruction model to optimally reconstruct the image by a population of neurons. In the image reconstruction model, **H** was estimated to directly optimize the image reconstruction considering the responses of multiple cells. By contrast, in the encoding model (or RF estimation), weights were estimated independently in each cell without considering the other cells’ responses. Because **H** was likely to be more optimized to represent images with multiple cells than **W** or RF, we chose the model scheme for the image reconstruction.

### Reconstruction performance against the number of features

In the analysis in Supplementary Fig. 5a–c, the cell-selection model was used for the image reconstruction. In the cell-selection model, each neuron participated in the reconstructions of a small number of features that were strongly correlated with the neuron’s responses. In the cell-selection model shown in Fig. 3 (the original model), the threshold for the correlation coefficient was selected based on the encoding model for each neuron (Fig. 3a, right panel). For each neuron, the threshold of the correlation coefficient was adjusted to increase (or decrease) the number of features for which each neuron participated in their reconstruction (0.1–20-fold change in the number of features per neuron relative to the original model, Supplementary Fig. 5a–c). For each fold change, the reconstruction model was trained with training data (i.e., weights and bias parameters were estimated in each fold change of the number of features), and the performance was estimated with test data using ten-fold CV as described above.

### Image reconstruction from a small number of cells

In the analyses shown in Figs. 4a–h and 5a, b, cells in each image were separated into responsive and remaining cells and sorted by their response amplitude in descending order (i.e., from highest to lowest response amplitude). Then, the cells were selected first from the responsive cells and then from the remaining cells for the addition (Fig. 4a–h) or dropping (Fig. 5a, b). The analyses only used data for images including at least ten responsive cells in Fig. 4a–h and at least five responsive cells in Fig. 5a–d.

In the image reconstruction from a subset of cells for each image (Figs. 4–6), the weights of the cell-selection model (${\mathbf{H}} = [{\mathbf{H}}^1; \ldots ;{\mathbf{H}}^{1248}]$, *f* × N_cell_) were scaled because **H** was estimated by more cells than the cells used during the cell addition or removal.

9 $${\mathbf{F}}^\prime = a^\prime {\mathbf{H}}\,{\mathbf{R}}^\prime + {\mathbf{c}}$$

where **F**′ (*f* × N_trial_) is the matrix of all reconstructed feature values, **H** and **c** ([$= [c^1; \ldots ;c^{1248}],$ *f* × 1) are the weight and bias matrices of the cell-selection model in Eq. (8), respectively, **R**′ is a response matrix that includes a subset of cells used for each image reconstruction (i.e., the responses of non-selected cells were set to 0), and *a*′is a free parameter that is obtained to minimize the sum of squared error between the original and reconstructed feature values across all features and stimuli of the training dataset in each CV: $\sum ({\mathbf{F}}^\prime -{\mathbf{F}})^2$ (**F**: a matrix of features of the regression target). Because *a*′ is common across all features, this scaling did not change the weight pattern of the cell-selection model. Then, images were reconstructed from **F**′ using Eq. (6) as described above. In the reconstruction from a subset of cells (Figs. 4–6), *a*′ (i.e., weights, *a*′ × **H**) was estimated independently for each subset of cells, and a different set of cells was used for each image.

### Robustness of image reconstruction against cell drop

In the analysis of robustness (Fig. 5c–f), a representation area for each cell was determined using the *z*-scored reverse filter (sum of weights × Gabor filters). The representation area was defined as a cluster of pixels whose absolute *z*-scores were >1.5 and whose contours were smoothed (e.g., red contours in Fig. 5c and Supplementary Fig. 6a). If multiple areas were obtained, the largest was used. Then, using the representation area, the overlap index was computed between responsive cells for each stimulus; overlap index = (A ∩ B)/(A ∪ B), where (A ∩ B) is the overlapping representation area between cell A and cell B, and (A ∪ B) is a combined representation area between cell A and cell B (Supplementary Fig. 6a). Using the overlap index, a set of overlapping cells was selected for each responsive cell; the overlapping cells consisted of one responsive cell (the reference cell) and the responsive cells that overlapped with the reference cell (overlap index > 0.2). This analysis did not care whether other overlapping cells overlapped with each other or with other non-selected cells.

To evaluate the effects of cell drop, cells were randomly removed from among the overlapping cells, and the reconstructed image was computed after each cell was dropped. The reference cell was initially removed, and then other remaining overlapping cells were removed in each cell drop sequence. Changes in the reconstructed images were estimated by quantifying the pixel-to-pixel correlation (R) of a local part of the image. The local part of the image was determined as the representation area of the reference cell that was overlapped by the area of at least one of the other overlapping cells (overlapping area in Fig. 5c). This random dropping of overlapping cells was repeated 120 times, and the results were averaged across the random orders for each reference cell. All responsive cells were used once as the reference cell for each stimulus image. This analysis only used data for images that included at least five responsive cells and sets of overlapping cells that included at least five overlapping cells.

### Across-trial similarity and variability

To estimate the reliability of the reconstructed images (or response patterns) across trials, two measures were used: across-trial similarity and across-trial variability. For the across-trial similarity of the reconstructed images (or response patterns; Fig. 6c), Pearson’s correlations between single-trial reconstructed images (or response patterns) and their trial average were computed and averaged across trials.

For the across-trial variability (Fig. 6d–g), the normalized squared error between single-trial images (or response patterns) and trial-averaged images (or response patterns) were computed using the following equation,

10 $${\mathrm{Across}} {\hbox{-}} {\mathrm{trial}}\,{\mathrm{variability}} = \frac{{\mathop {\sum }\nolimits_t \mathop {\sum }\nolimits_u ({\mathbf{A}}_{t,u} - {\mathbf{A}}_u)^2/({\mathrm{N}}_u({\mathrm{N}}_{{\mathrm{trial}}} - 1))}}{{{\mathrm{N}}_{{\mathrm{trial}}}\mathop {\sum }\nolimits_u ({\mathbf{A}}_u - {\bar{\mathbf{A}}})^2/({\mathrm{N}}_u - 1)}}$$

**A**_*t*,*u*_: single-trial reconstructed image or response pattern, *t*: trial number, *u*: pixel or cell number, **A**_*u*_: trial-averaged reconstructed image or response pattern, ${\bar{\mathbf{A}}}:$ mean of **A**_*u*_ across pixels or cells, N_*u*_: number of pixels or cells, N_trial_: number of trials, ∑: summation across trials, and ∑: summation across pixels or cells. In this variability measure, we first computed the squared error between **A**_*t*,*u*_ and **A**_*u*_ (i.e., $(\mathop {\sum}\nolimits_t {\mathop {\sum}\nolimits_u {({\mathbf{A}}_{t,u} - {\mathbf{A}}_u)^2)/{\mathrm{N}}_{{\mathrm{trial}}}} }$, which reflects deviations of single-trial images or responses from that of trial average) and averaged across trials. However, this squared error could be larger when the pixel intensities are larger in the reconstructed image or when the response amplitudes are larger in the cell population. To control this problem, this squared error was normalized by another squared error between **A**_*u*_ and ${\bar{\mathbf{A}}}$ (i.e., $\mathop {\sum}\nolimits_u {\left( {{\mathbf{A}}_u - {\bar{\mathbf{A}}}} \right)^2}$, which reflects deviations of pixels’ intensity or cells’ responses from the mean value across pixels or cells in the trial-averaged data). To correct differences between the number of pixels and cells (i.e., the number of degrees of freedom), each squared error was divided by the degree of freedom (${\mathrm{N}}_u\left( {{\mathrm{N}}_{{\mathrm{trial}}} - 1} \right)$ for numerator and (N_*u*_ − 1) for denominator), following the correction of squared error by degree of freedom in ANOVA wherein pixels or cells correspond to groups and trials correspond to samples. This across-trial variability equals the inverse of the *F*-value in the ANOVA.

In the analysis depicted in Fig. 6f, g, the overlapping cells were selected as described above for each responsive cell (i.e., the reference cell; see the section titled “Robustness of image reconstruction against cell drop”). The reference cell was initially selected, and then other overlapping cells were randomly selected for a set of cells that were used for image reconstruction (the sequences of random cell selection were repeated 200 times.). The image was reconstructed from the subset of overlapping cells, and across-trial variability of a local part (i.e., overlapping area) of the reconstructed image was computed for each subset of cells. Only data for images that contained at least five responsive cells were used in the analyses in Fig. 6.

### Noise correlation

In the analysis depicted in Supplementary Fig. 7, the noise correlation was computed using the responses across stimuli. Evoked responses to each stimulus image were transformed to *z*-scores and collected across stimuli in each cell. Then, Pearson’s correlation coefficient was computed between the collected responses in a cell pair and used as the noise correlation. To remove the noise correlation, responses to each stimulus were shuffled across trials independently in each cell. Using the shuffled data, an image reconstruction model was obtained as described above for the analyses in Fig. 6g, Supplementary Fig. 7g–i, p–r, and Supplementary Fig. 9f.

### Capacity for image representation of encoded features

The analysis in Fig. 7e–g illustrates whether the features encoded by responsive cells could represent images as a basis function independent of actual neural responses. If the features encoded by responsive neurons can represent any image, a set of features of a given image (**F**) will be linearly regressed by the weights of the responsive cells in the cell-selection model, **H** ($= [{\mathbf{H}}^1; \ldots ;{\mathbf{H}}^{1248}]$, *f*  ×  N_cell_; **H**^*k*^ in Eq. (8)),

11 $${\mathbf{F}} = {\mathbf{H}}\,{\mathbf{B}} + {\mathbf{d}} + {\mathbf{e}}$$

where **B** and **d** are free parameters that are calculated to minimize the sum of the squared error, $\sum ({\mathbf{F}}-({\mathbf{H}}\,{\mathbf{B}} + {\mathbf{d}}))^2$, and **e** is an error term. **F** was selected from a test dataset, and **H** was obtained from a training dataset in the ten-fold CV. The fitting was evaluated by calculating the fitting error (Error) on the image space as follows:

12 $${\mathbf{I}}^\prime \, = \,{\mathbf{G}}_{{\mathrm{rev}}}\,{\mathbf{F}}$$

13 $${\hat{\mathbf{I}}} = {\mathbf{G}}_{{\mathrm{rev}}}\,{\hat{\mathbf{F}}}$$

14 $${\hat{\mathbf{F}}} = {\mathbf{H}}\,{\mathbf{B}} + {\mathbf{d}}$$

15 $${\mathrm{Error}} = {\sum} {\left( {{\mathbf{I}}^\prime - {\hat{\mathbf{I}}}} \right)^2} /{\sum} {\left( {{\mathbf{I}}^\prime - {\mathbf{I}}_{{\mathrm{mean}}}^\prime } \right)^2} \times 100$$

where **G**_rev_ is the Gabor filter matrix used for reconstruction (Eq. (6)), and ${\mathbf{I}}_{{\mathrm{mean}}}^\prime$ is the mean pixel intensity of **I**′. Thus, this analysis estimates how well the features of individual neurons in a local population could represent the image features independent of actual neuronal activity. In other words, this analysis estimates the upper-bound capacity of a local population to represent any image with an ideal combination of cell features (with parameters **B** and **d**).

### Effects of locomotion state on image reconstruction

Because the awake mice were not trained to run, they often stayed calmly during imaging. In the analyses shown in Supplementary Fig. 11, we included only data from two planes in one mouse (Thy1-GCaMP6s mouse) that ran relatively frequently. Furthermore, we used only data for images that contained at least five responsive cells, four running trials, and four resting trials (80 image cases, *n* = 295 responsive cells). The running modulation index (RMI) for each cell was defined as follows: RMI = (R_run_ − R_rest_)/(R_run_ + R_rest_), where R_run_ and R_rest_ were the mean evoked responses during running and resting, respectively. RMI was computed in each responded image and averaged across images in each cell. Image reconstruction was performed using data with both conditions, and the performances were collected separately in each condition (Supplementary Fig. 11d, e).

### Statistical analyses

All data are presented as the median and 25th–75th percentiles unless indicated otherwise. The significant level was set to 0.05, with the exception of the criteria of significant visual response (0.01). When more than two groups were compared, the significant level was adjusted with the Bonferroni correction except for the visually responsive cell analysis. Two-sided test was used in all analyses. The experiments were not performed in a blind manner. The sample sizes were not predetermined by any statistical methods but are comparable to the sample size of other reports in the field.

### Reporting summary

Further information on research design is available in the Nature Research Reporting Summary linked to this article.

## Supplementary information

Supplementary Information

Reporting Summary

## Data Availability

The datasets of the current study are available from the corresponding authors on reasonable request. The source data underlying Fig. 1e–k, 2g, i, j, 3f, g, 4d, e, g, h, j, k, 5b, f, 6c, d, g, 7d, g, and 8e–h, and Supplementary Figs. 1a–d, 2b–g, 3b–d, g, h, j, k, 4c, d, 5b, c, e, f, 6b–d, 7a–c, g–i, j–l, p–r, 8a–c, e–k, 9c, d, f, 10, and 11 are provided as a Source Data file.

## Source data

Source Data

## Code availability

The codes used to analyze the data of current study are available from the corresponding authors on reasonable request.

## References

[1] Rolls ET, Tovee MJ (1995). Sparseness of the neuronal representation of stimuli in the primate temporal visual cortex. J. Neurophysiol..

[2] Vinje WE, Gallant JL (2000). Sparse coding and decorrelation in primary visual cortex during natural vision. Science.

[3] Weliky M, Fiser J, Hunt RH, Wagner DN (2003). Coding of natural scenes in primary visual cortex. Neuron.

[4] Olshausen BA, Field DJ (2004). Sparse coding of sensory inputs. Curr. Opin. Neurobiol..

[5] Froudarakis E (2014). Population code in mouse V1 facilitates readout of natural scenes through increased sparseness. Nat. Neurosci..

[6] Yen SC, Baker J, Gray CM (2007). Heterogeneity in the responses of adjacent neurons to natural stimuli in cat striate cortex. J. Neurophysiol..

[7] Yao H, Shi L, Han F, Gao H, Dan Y (2007). Rapid learning in cortical coding of visual scenes. Nat. Neurosci..

[8] Tolhurst DJ, Smyth D, Thompson ID (2009). The sparseness of neuronal responses in ferret primary visual cortex. J. Neurosci..

[9] Willmore BD, Mazer JA, Gallant JL (2011). Sparse coding in striate and extrastriate visual cortex. J. Neurophysiol..

[10] Field DJ (1994). What Is the goal of sensory coding. Neural Comput.

[11] Jones JP, Palmer LA (1987). An evaluation of the two-dimensional Gabor filter model of simple receptive fields in cat striate cortex. J. Neurophysiol..

[12] Olshausen BA, Field DJ (1996). Emergence of simple-cell receptive field properties by learning a sparse code for natural images. Nature.

[13] Bell AJ, Sejnowski TJ (1997). The “independent components” of natural scenes are edge filters. Vis. Res..

[14] Tang, S., et al. Large-scale two-photon imaging revealed super-sparse population codes in the V1 superficial layer of awake monkeys. *Elife* **7**, e33370 (2018). 10.7554/eLife.33370 PMC5953536 29697371

[15] Stanley GB, Li FF, Dan Y (1999). Reconstruction of natural scenes from ensemble responses in the lateral geniculate nucleus. J. Neurosci..

[16] Miyawaki Y (2008). Visual image reconstruction from human brain activity using a combination of multiscale local image decoders. Neuron.

[17] Naselaris T, Prenger RJ, Kay KN, Oliver M, Gallant JL (2009). Bayesian reconstruction of natural images from human brain activity. Neuron.

[18] Nishimoto S (2011). Reconstructing visual experiences from brain activity evoked by natural movies. Curr. Biol..

[19] Horikawa T, Tamaki M, Miyawaki Y, Kamitani Y (2013). Neural decoding of visual imagery during sleep. Science.

[20] Doi E, Lewicki MS (2005). Sparse coding of natural images using an overcomplete set of limited capacity units. Adv. Neural Inf. Process. Syst..

[21] Smith SL, Hausser M (2010). Parallel processing of visual space by neighboring neurons in mouse visual cortex. Nat. Neurosci..

[22] Bonin V, Histed MH, Yurgenson S, Reid RC (2011). Local diversity and fine-scale organization of receptive fields in mouse visual cortex. J. Neurosci..

[23] Kampa BM, Roth MM, Gobel W, Helmchen F (2011). Representation of visual scenes by local neuronal populations in layer 2/3 of mouse visual cortex. Front. Neural Circuits.

[24] Ko H (2011). Functional specificity of local synaptic connections in neocortical networks. Nature.

[25] Marshel JH, Garrett ME, Nauhaus I, Callaway EM (2011). Functional specialization of seven mouse visual cortical areas. Neuron.

[26] Miller JeK, Ayzenshtat I, Carrillo-Reid L, Yuste R (2014). Visual stimuli recruit intrinsically generated cortical ensembles. Proc. Natl Acad. Sci. USA.

[27] Rikhye RV, Sur M (2015). Spatial correlations in natural scenes modulate response reliability in mouse visual cortex. J. Neurosci..

[28] Olshausen BA, Field DJ (2005). How close are we to understanding V1?. Neural. Comput..

[29] Shoham S, O’Connor DH, Segev R (2006). How silent is the brain: is there a “dark matter” problem in neuroscience?. J. Comp. Physiol. A Neuroethol. Sens. Neural. Behav. Physiol..

[30] Yoshida, T. & Ohki, K. *Visual image reconstruction from neuronal activities in the mouse primary visual cortex*. *Program No. 415.17. 2015 Neuroscience Meeting Planner.* (Chicago, IL: Society for Neuroscience, 2015).

[31] Yoshida, T. & Ohki, K. Robust representation of natural images by sparse and variable population of active neurons in visual cortex. Preprint at https://www.biorxiv.org/content/10.1101/300863v2 (2018). 10.1038/s41467-020-14645-x PMC7018721 32054847

[32] Smyth D, Willmore B, Baker GE, Thompson ID, Tolhurst DJ (2003). The receptive-field organization of simple cells in primary visual cortex of ferrets under natural scene stimulation. J. Neurosci..

[33] Ko H (2013). The emergence of functional microcircuits in visual cortex. Nature.

[34] Cossell L (2015). Functional organization of excitatory synaptic strength in primary visual cortex. Nature.

[35] Niell CM, Stryker MP (2008). Highly selective receptive fields in mouse visual cortex. J. Neurosci..

[36] Shadlen MN, Newsome WT (1994). Noise, neural codes and cortical organization. Curr. Opin. Neurobiol..

[37] Zohary E, Shadlen MN, Newsome WT (1994). Correlated neuronal discharge rate and its implications for psychophysical performance. Nature.

[38] Moreno-Bote R (2014). Information-limiting correlations. Nat. Neurosci..

[39] Cohen MR, Kohn A (2011). Measuring and interpreting neuronal correlations. Nat. Neurosci..

[40] Chen TW (2013). Ultrasensitive fluorescent proteins for imaging neuronal activity. Nature.

[41] Dana H (2014). Thy1-GCaMP6 transgenic mice for neuronal population imaging in vivo. PLoS One.

[42] Madisen L (2010). A robust and high-throughput Cre reporting and characterization system for the whole mouse brain. Nat. Neurosci..

[43] Taniguchi H (2011). A resource of Cre driver lines for genetic targeting of GABAergic neurons in cerebral cortex. Neuron.

[44] Niell CM, Stryker MP (2010). Modulation of visual responses by behavioral state in mouse visual cortex. Neuron.

[45] Ayaz A, Saleem AB, Scholvinck ML, Carandini M (2013). Locomotion controls spatial integration in mouse visual cortex. Curr. Biol..

[46] Greenberg DS, Houweling AR, Kerr JN (2008). Population imaging of ongoing neuronal activity in the visual cortex of awake rats. Nat. Neurosci..

[47] Rehn M, Sommer FT (2007). A network that uses few active neurones to code visual input predicts the diverse shapes of cortical receptive fields. J. Comput. Neurosci..

[48] Olshausen BA, Cadieu CF, Warland DK (2009). Learning real and complex overcomplete representations from the statistics of natural images. SPIE Optical Eng. + Appl..

[49] Olshausen, B.A. Highly overcomplete sparse coding. In *Proc. 2013 Society of Photo-Optical Instrumentation Engineers (SPIE), Electronic Imaging* **8651**, 86510S https://www.spiedigitallibrary.org/conference-proceedings-of-spie/8651/86510S/Highly-overcomplete-sparse-coding/10.1117/12.2013504.full?SSO=1&tab=ArticleLink (2013).

[50] Stringer C, Pachitariu M, Steinmetz N, Carandini M, Harris KD (2019). High-dimensional geometry of population responses in visual cortex. Nature.

[51] Srivastava N, Hinton G, Krizhevsky A, Sutskever I, Salakhutdinov R (2014). Dropout: a simple way to prevent neural networks from overfitting. J. Mach. Learn. Res..

[52] Nimmerjahn A, Kirchhoff F, Kerr JN, Helmchen F (2004). Sulforhodamine 101 as a specific marker of astroglia in the neocortex in vivo. Nat. Methods.

[53] Ohki K, Chung S, Ch’ng YH, Kara P, Reid RC (2005). Functional imaging with cellular resolution reveals precise micro-architecture in visual cortex. Nature.

[54] Hagihara KM, Murakami T, Yoshida T, Tagawa Y, Ohki K (2015). Neuronal activity is not required for the initial formation and maturation of visual selectivity. Nat. Neurosci..

[55] Mank M (2008). A genetically encoded calcium indicator for chronic in vivo two-photon imaging. Nat. Methods.

[56] van Hateren JH, van der Schaaf A (1998). Independent component filters of natural images compared with simple cells in primary visual cortex. Proc. Biol. Sci..

[57] Olmos A, Kingdom FA (2004). A biologically inspired algorithm for the recovery of shading and reflectance images. Perception.

[58] Fei-Fei, L., Fergus, R. & Perona, P. Learning generative visual models from few training examples: an incremental Bayesian approach tested on 101 object categories. In *Proc.2004 Conference on Computer Vision and Pattern Recognition Workshop*, 178–178 (2004).

[59] Peirce JW (2008). Generating stimuli for neuroscience using PsychoPy. Front. Neuroinform..

[60] Stahl JS, van Alphen AM, De Zeeuw CI (2000). A comparison of video and magnetic search coil recordings of mouse eye movements. J. Neurosci. Methods.

[61] Kerlin AM, Andermann ML, Berezovskii VK, Reid RC (2010). Broadly tuned response properties of diverse inhibitory neuron subtypes in mouse visual cortex. Neuron.

[62] Treves A, Rolls ET (1991). What determines the capacity of autoassociative memories in the brain?. Netw. Comput. Neural Syst..

[63] Lee TS (1996). Image representation using 2D gabor wavelets. IEEE Trans. Pattern Anal. Mach. Intell..

[64] Kay KN, Naselaris T, Prenger RJ, Gallant JL (2008). Identifying natural images from human brain activity. Nature.

[65] Bishop, C.M. *Pattern Recognition and Machine Learning (Information Science and Statistics)* (Springer-Verlag, New York, Inc. 2006).

