# Supplementary material for: Natural images are reliably represented by sparse and variable populations of neurons in visual cortex
Source: Nat Commun. 2020 Feb 13;11:872. doi: 10.1038/s41467-020-14645-x (PMC7018721; doi:10.1038/s41467-020-14645-x)
Supplement: Supplementary file 1 — Supplementary Information [file 41467_2020_14645_MOESM1_ESM.pdf]

Supplementary information

Natural images are reliably represented by sparse and variable population of neurons in visual cortex

Yoshida and Ohki

Contents

Supplementary Figure 1.

Supplementary Figure 2.

Supplementary Figure 3.

Supplementary Figure 4.

Supplementary Figure 5.

Supplementary Figure 6.

Supplementary Figure 7.

Supplementary Figure 8.

Supplementary Figure 9.

Supplementary Figure 10.

Supplementary Figure 11.

Supplementary References

Supplementary Figure 1. Responsive rates for each image and false positive rates

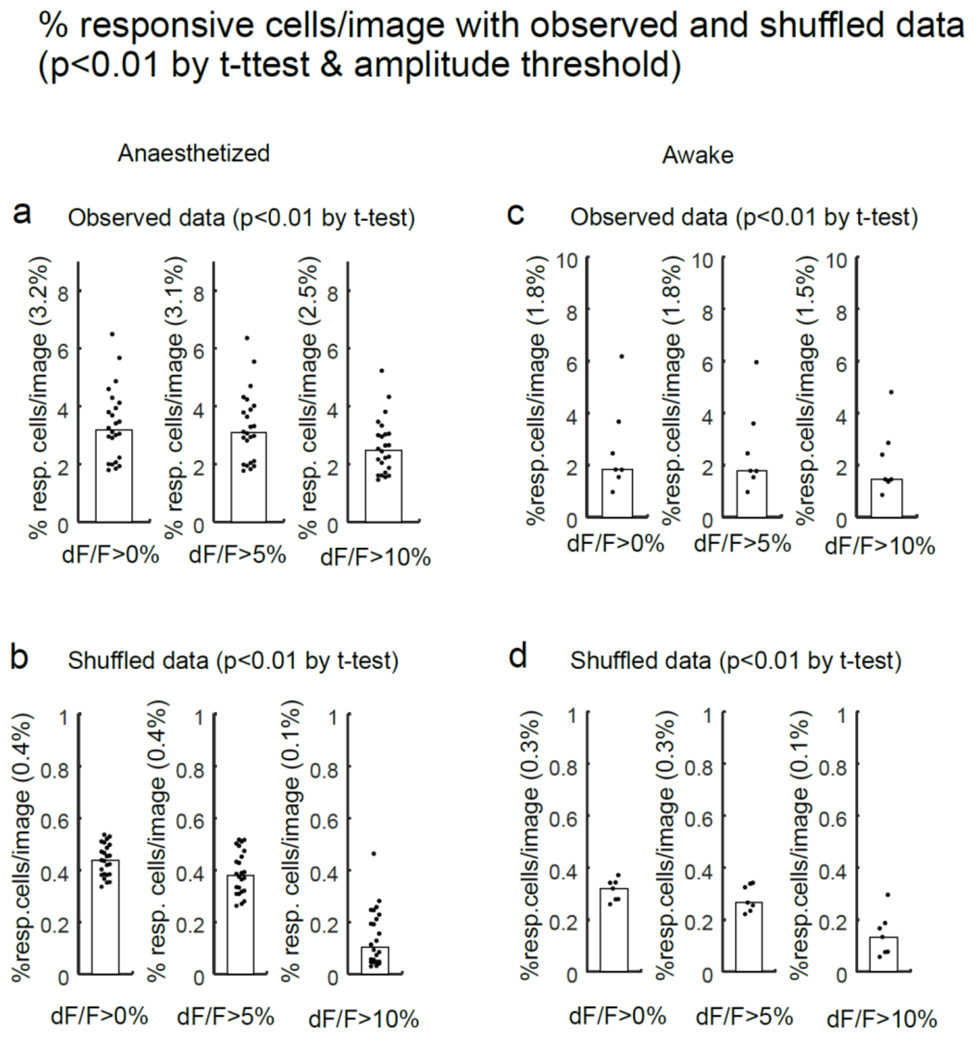

Supplementary Figure 1. Responsive rates for each image and false positive rates

a and **b.** Percentages of responsive cells per image in anaesthetized mice. In each responsive cell identified using ANOVA (p<0.01), responsiveness for each image was determined using a t-test (p <0.01, baseline vs. stimulus activity) and evoked response amplitudes. **a**. Percentages of responsive cells per image obtained from observed data with three evoked response thresholds (0%, 5%, and 10%). **b**. False positive rate estimated with label-shuffled data. The 10% threshold resulted in a small fraction of the false positive rate relative to the responsive rate obtained from the original data.
c and **d**. Percentages of responsive cells per image in awake mice. Same as in (**a**) and (**b**), except for awake data
Source data are provided as a Source Data file.

Supplementary Figure 2. Prediction performance of the encoding model

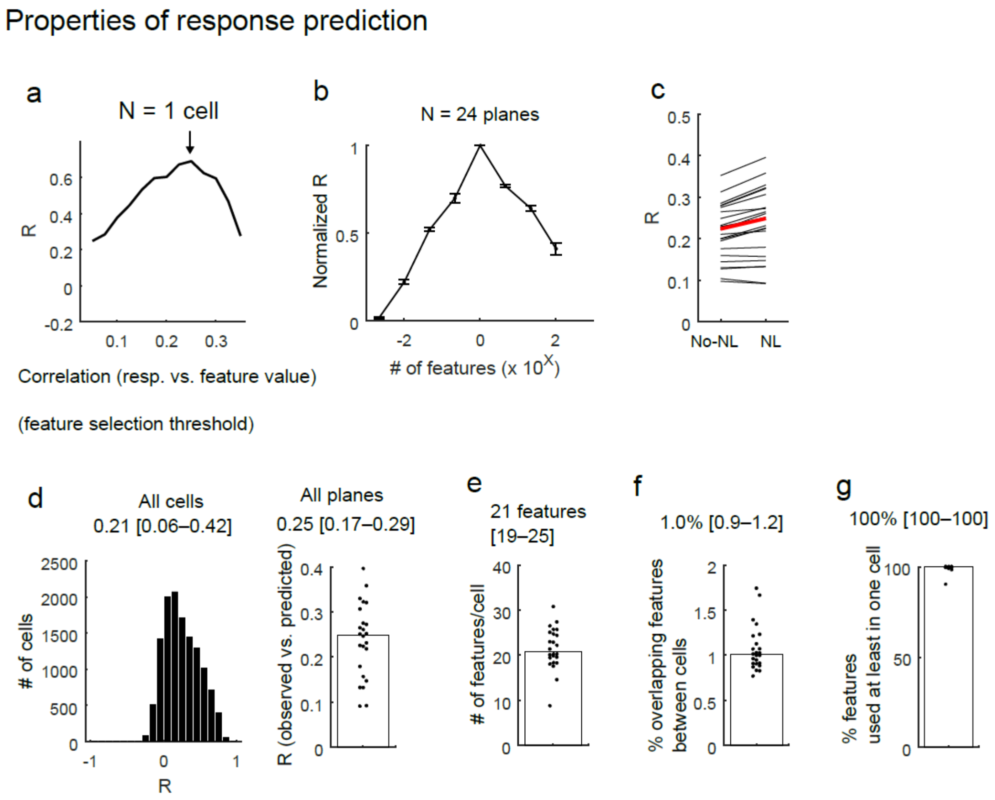

Supplementary Figure 2. Prediction performance of the encoding model

a. Effect of feature selection on the response prediction performance of the encoding model in an example neuron. The response prediction performances (correlation coefficients between the observed and predicted responses, R) are plotted against the threshold values of the feature selection. First, Pearson's correlation coefficient between each feature value and response was computed. Then, the features with correlation coefficients greater than the preset threshold values (x-axis) were used for the regression analysis of the encoding model. The threshold value for the final model was selected from the values to maximize the response prediction for each neuron (arrow).
b. Normalized prediction performance against the number of features used for the encoding model. The data plotted in (**a**) were normalized by the maximal performance (y-axis) and re-plotted against the number of features used in the model of each threshold (x-axis). Data were collected across all cells in each plane, resampled and averaged in each bin (x-axis in the plot). The plotted curve was averaged across planes. Means ± S.E.M.s are shown (n = 24 planes).
c. Response prediction performances of the encoding models with and without a nonlinear scaling step. R: correlation coefficient between observed and predicted responses. No-NL: model without the nonlinear scaling step. NL: model with the non-linear scaling step. No-NL: 0.22 [0.17–0.27]. NL: 0.25 [0.17–0.29]. $P = 1.1 \times 10^{-4}$ by signed-rank test (n = 24 planes).
d. Distributions of the response prediction performances for all cells (0.21 [0.06–0.42], n = 12,755 cells, left) and for all planes (0.25 [0.17–0.29], n = 24 planes, right).
e. Number of features encoded by each cell (21 [19–25] features, n = 24 planes).
f. Percentages of overlapping features between cells (1.0% [0.9–1.2%], n = 24 planes).
g. Percentages of features that were used for the response prediction of at least one cell in a population (100% [100–100%], n = 24 planes).
Source data are provided as a Source Data file.

Supplementary Figure 3. Properties of Gabor features encoded by individual cells.

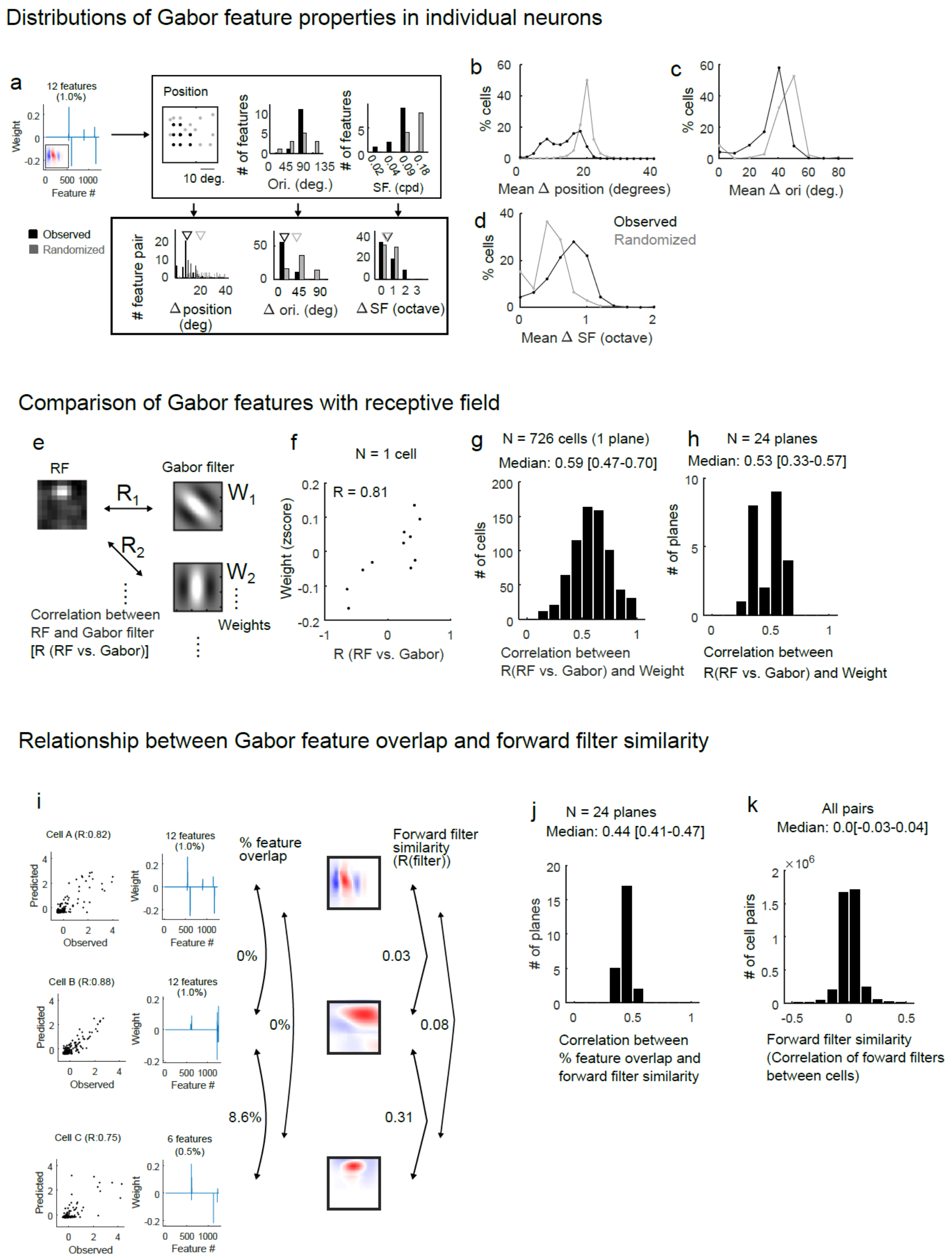

Supplementary Figure 3. Properties of Gabor features encoded by individual cells.

a–d. Properties of Gabor features encoded by individual cells.
a. Schematic of the analysis. Upper panels: Features encoded by each cell were analysed in terms of retinotopy (position, **b**), orientation (Ori, **c**) and spatial frequency (SF, **d**). Lower panels: similarities among features in each cell were evaluated by computing differences in positions, orientation, and spatial frequency between features. Mean values in each cell were collected across all cells and plotted in (**b–d**, observed). As a control, the labels of Gabor features were randomized in each cell while preserving the number of features (randomized). Relatively narrow distributions of randomized data reflect the bias of the original Gabor filter properties (e.g., slightly more Gabor filters are located at the centre positions, see Fig. 2b).

e–h. Relationship between the weights of the Gabor features and the RF structure.
e and **f**. Schematics of the analysis. (**e**) In each cell, the RF structure was determined using the pseudoinverse method (see Methods), and pixel-to-pixel Pearson's correlation coefficients between the RFs and Gabor filters were computed (R1 and R2 in (**e**); R (RF vs. Gabor)). (**f**) Then, Pearson's correlation coefficients between the R (RF vs. Gabor) and weight values (W1 and W2 in (**e**)) was computed for each cell.
g and **h**. Distributions of the correlation coefficient between R (RF vs. Gabor) and weights. Distributions for all cells in the example plane (**g**) and for all planes (**h**).

i–k. Relationship between the percentage of overlapping features and the forward filter similarity in a cell pair.
i. Examples of response prediction performances (left panels), weights (centre) and forward filters (right panels) of the three cells (cells A, B and C). The red and blue colours of the forward filter indicate positive and negative values, respectively.
j. Distribution of the correlation coefficient between the % overlapping feature and the absolute value of the forward filter similarity (n = 24 planes).
k. Diverse structure of the forward filters. The similarity of the forward filter was relatively small for all pairs (median: 0.0), which indicated the diverse structure of the forward filters.
Source data are provided as a Source Data file.

Supplementary Figure 4. Image reconstruction performances with and without nested CV

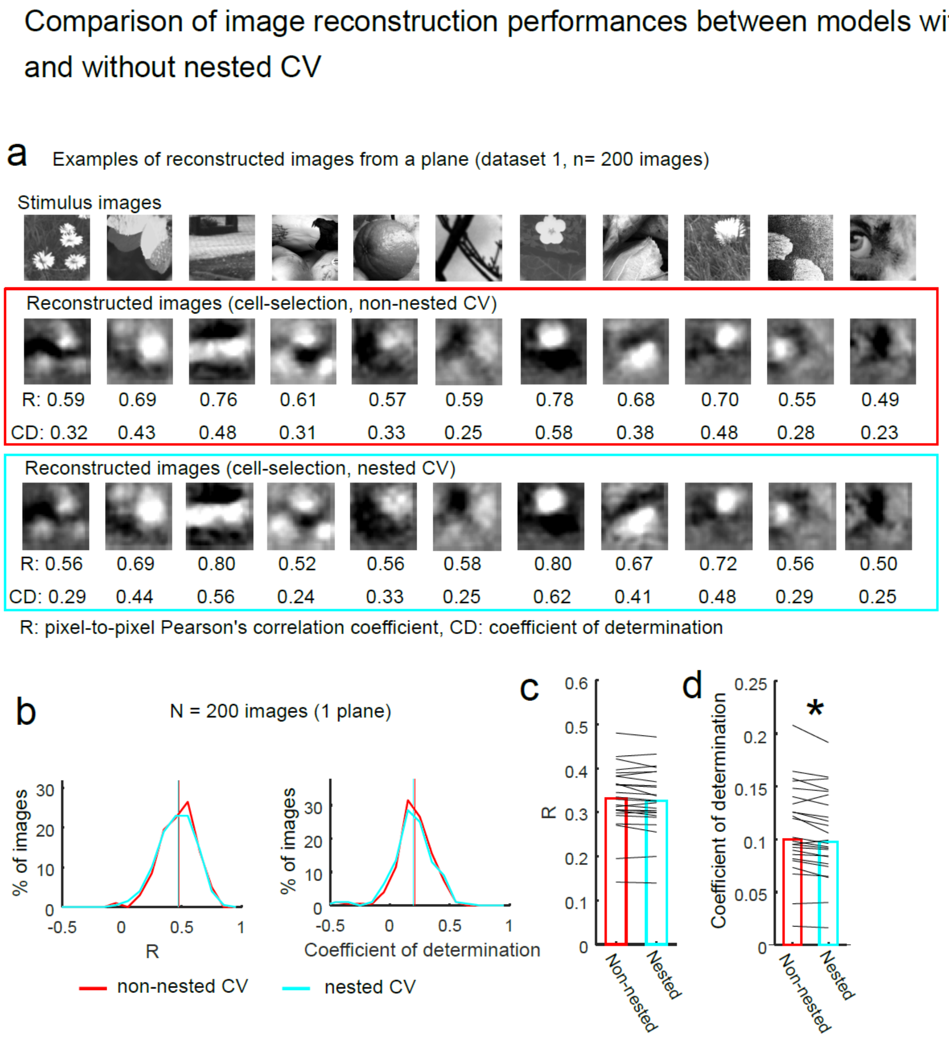

Supplementary Figure 4. Image reconstruction performances with and without nested CV

a. Examples of reconstructed images from main datasets (dataset 1; 200 images). Stimulus images (top), images that were reconstructed using the cell-selection model without nested CV (non-nested CV, middle, also shown in Fig. 3c in the main text) and using the cell-selection model with nested CV (nested CV, bottom) are shown. Each reconstructed image was averaged across trials. The reconstruction performances (R and coefficient of determination, CD) were computed for each trial, and trial-averaged performances are presented below each reconstructed image.
b. Distributions of R (left) and CD values (right) for the model without nested CV (red lines) and the model with nested-CV (cyan lines) in the example plane shown in Figs. 1 and 2 (n = 200 images reconstructed using 726 cells from a plane). Vertical lines indicate median values.
c and **d**. R (**c**) and CD (**d**) of dataset 1 across planes. *: p = 0.001 in (**d**) using the signed-rank test (n = 24 planes). The reconstruction performances of the cell-selection model with nested CV are similar to those with non-nested CV.
The stimulus images in (**a**) are adapted from the databases in supplementary references 1 and 2 with permission.
Source data are provided as a Source Data file.

Supplementary Figure 5. Reconstruction performances against the number of features per cell and spatial frequency components

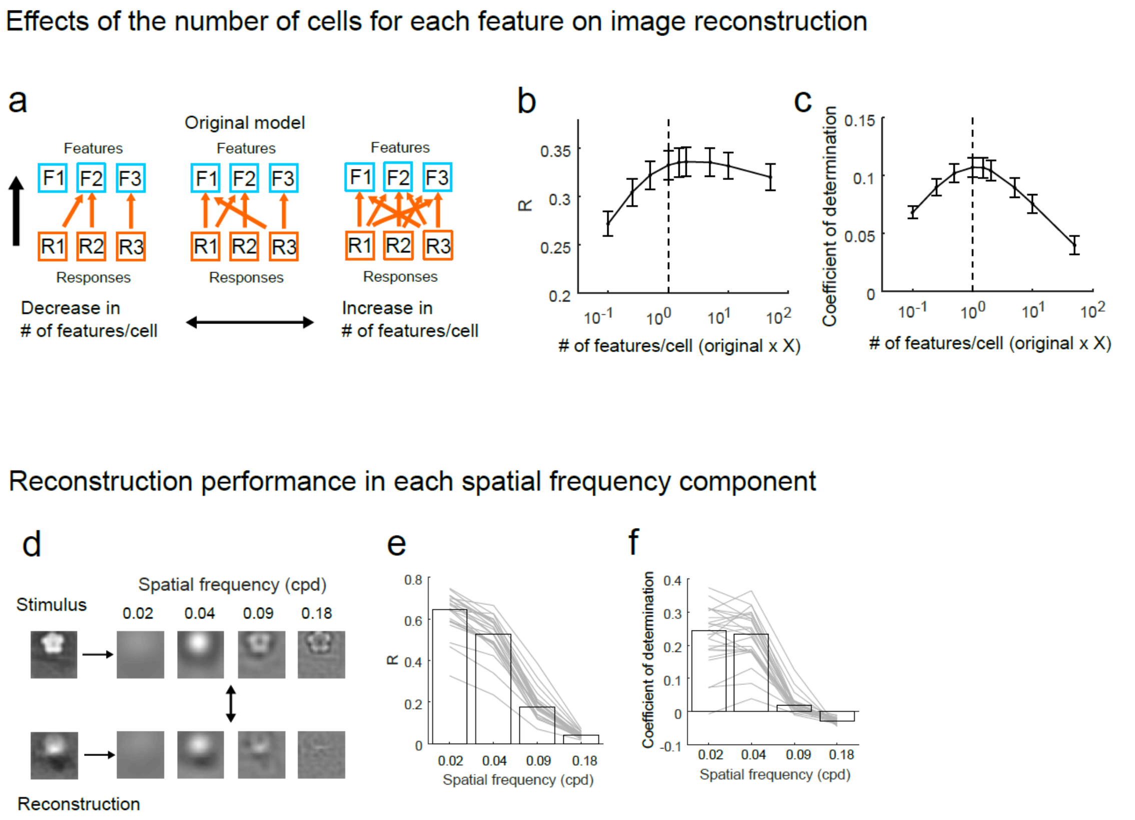

Supplementary Figure 5. Reconstruction performances against the number of features per cell and spatial frequency components

a. Schematic of the analysis. This analysis examined how the image reconstruction performance was affected by changing the number of features in which each cell participated in the reconstruction.
b and **c.** Reconstruction performances (R in **b,** and coefficient of determination in **c**) against the number of features per cell. The number of features in the original model (shown in the main text) is indicated by the dotted vertical line (x = original × 1) in each panel. The original model demonstrated nearly optimal performance, suggesting that how the individual neurons encode Gabor features is an important factor in image reconstruction and that the original model captures nearly optimal feature-cell assignment.
d–f. Reconstruction performance in each spatial frequency component by the cell-selection model. (**d**) Schematic of the analysis. Images were reconstructed using features for each spatial frequency. (**e** and **f**) The pixel-to-pixel correlation coefficient (R, **e**) and coefficient of determination (**f**) are plotted (n = 24 planes). Relatively low spatial frequency components of images were represented by V1 neurons. The stimulus image in (**d**) is adapted from the database in supplementary reference 1 with permission. Source data are provided as a Source Data file.

Supplementary Figure 6. Spatial overlap of reverse filters among responsive neurons

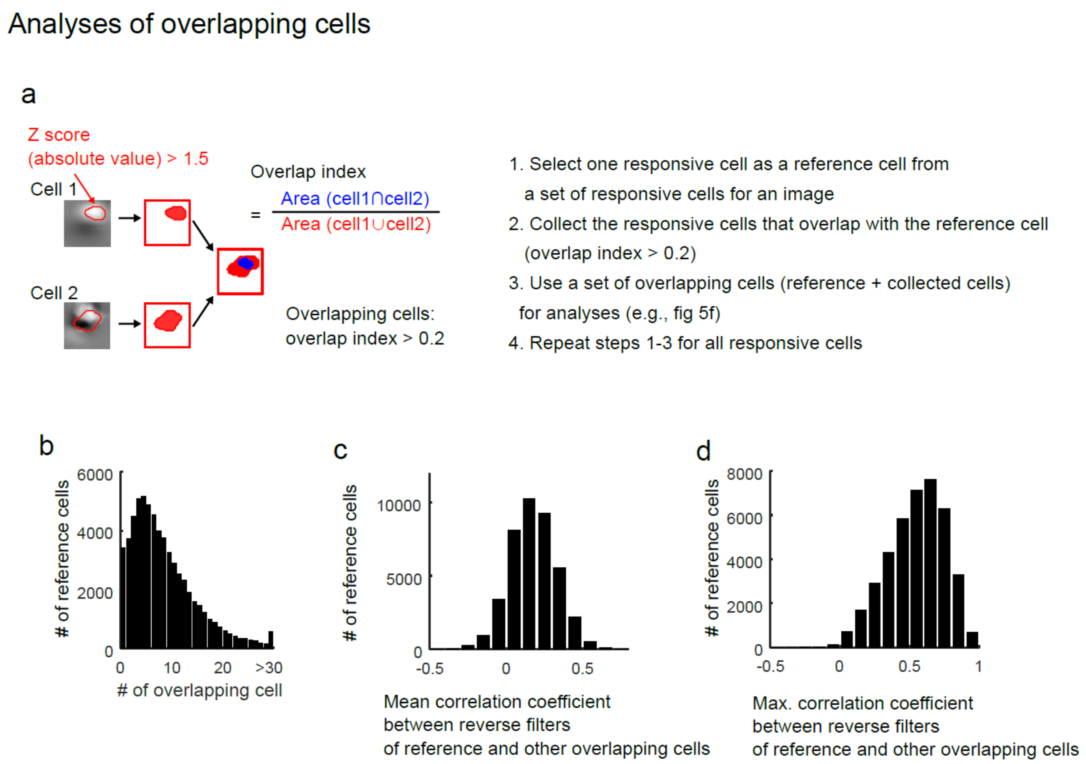

Supplementary Figure 6. Spatial overlap of reverse filters among responsive neurons

a. Spatial overlap of reverse filters. The reverse filter was transformed to z-scores, and the area in which the absolute z-score was greater than 1.5 was defined as a significant area (red contours in the left panels). The significant area was used to compute the overlap index. For each responsive cell (reference cell), the overlap index between the reference cell and other responsive cells was computed, and the responsive cells whose overlap indices were greater than 0.2 were collected to form a set of overlapping cells (reference and collected cells).
b. Distribution of the number of spatially overlapping cells for each responsive cell.
c. Distribution of the mean correlation coefficients between reverse filters for a reference cell and the other overlapping cells.
d. Distribution of maximal correlation coefficients between reverse filters for a reference cell and the other overlapping cells.
Source data are provided as a Source Data file.

Supplementary Figure 7. Relationship between noise correlation and reverse filter similarity

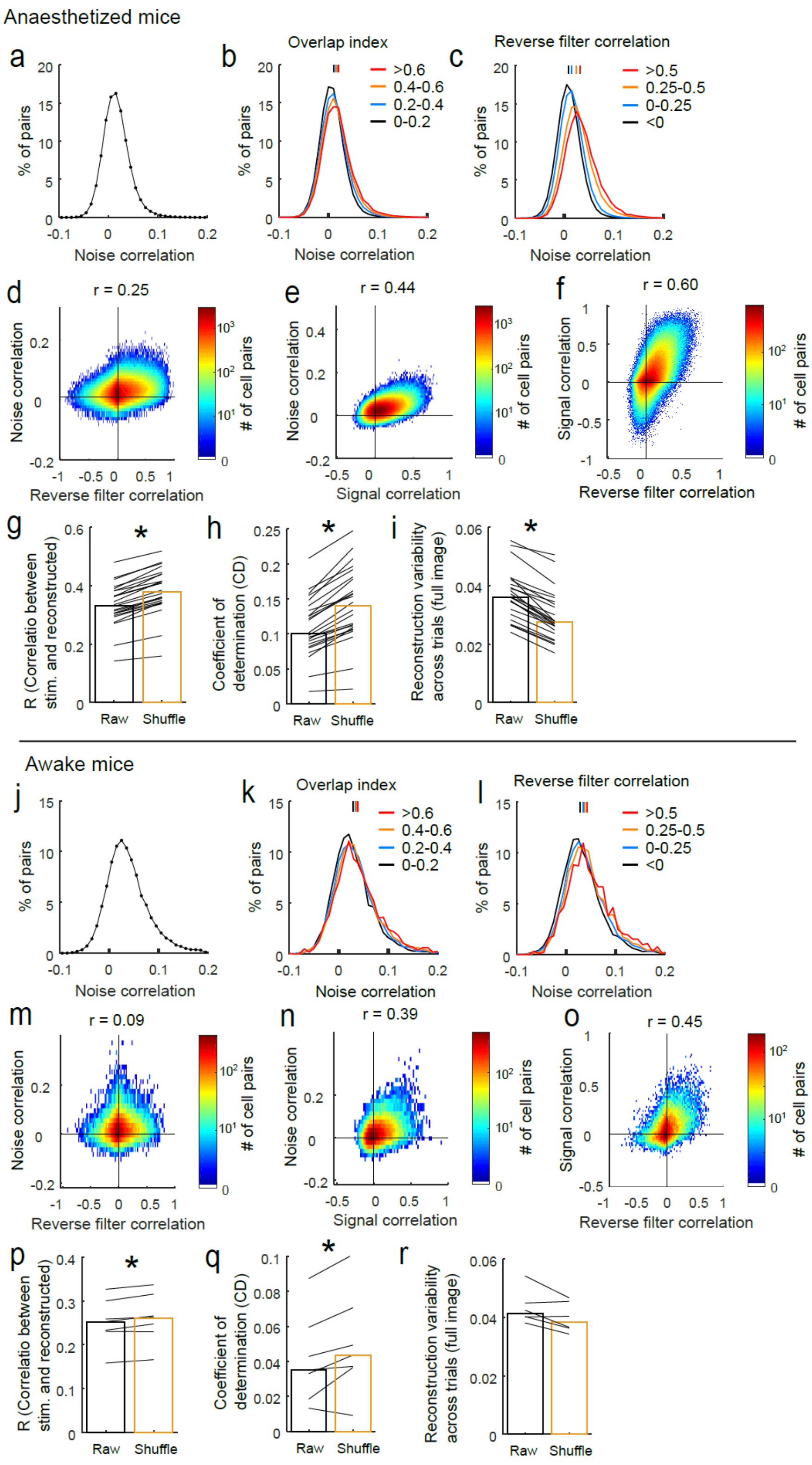

Supplementary Figure 7. Relationship between noise correlation and reverse filter similarity

a. Distribution of noise correlations from all responsive cell pairs (0.014 [-0.002–0.031]) in anaesthetized mice.
b and **c**. Distributions of noise correlations grouped by overlap index (**b**) and reverse filter similarity (pixel-to-pixel correlation of reverse filters, **c**). The distribution in (**a**) was divided into four groups based on the overlap index or the reverse filter correlations, and the distributions of the groups were plotted with different colours. Vertical lines over distribution curves indicate median values. In the analyses in this figure, only data for images that had at least five responsive cells were used.
d. Relationship between reverse filter and noise correlations.
e. Relationship between signal and noise correlations.
f. Relationship between reverse filter and signal correlations. (**d**–**f**) Data of responsive cell pairs were included. Colour indicates the number of cell pairs in each bin (bin size: 0.01).
g–i. Effect of noise correlation on image reconstruction performances (R in **g** and CD in **h**) and across-trial variability (**i**). Shuffle: trial-shuffled data. *: $p = 1.8\times10^{-5}$ (**g**), $p= 1.8\times10^{-5}$ (**h**) and $p=1.8\times10^{-5}$ (**i**) by signed-rank test (n = 24 planes).
j. Distribution of noise correlations from all responsive cell pairs (0.03 [0.009–0.06]) in awake mice.
k and **l**. Distribution of noise correlations grouped by overlap index (**k**) or reverse filter correlation (**l**). Same as in **b**and **c** except for awake data.
m–o. Relationship among reverse filter, noise, and signal correlations in awake mice. Same as in **d–f** except for awake data and bin size (0.02).
p–r. Effect of noise correlations on image reconstruction performances (**p** and **q**) and across-trial variability (**r**) in awake mice. *: p = 0.03 (**p**), p= 0.047 (**q**) by signed-rank test (n = 7 planes).
Source data are provided as a Source Data file.

Supplementary Figure 8. Response properties, response prediction and image reconstruction in awake mice (related to Fig. 1–5)

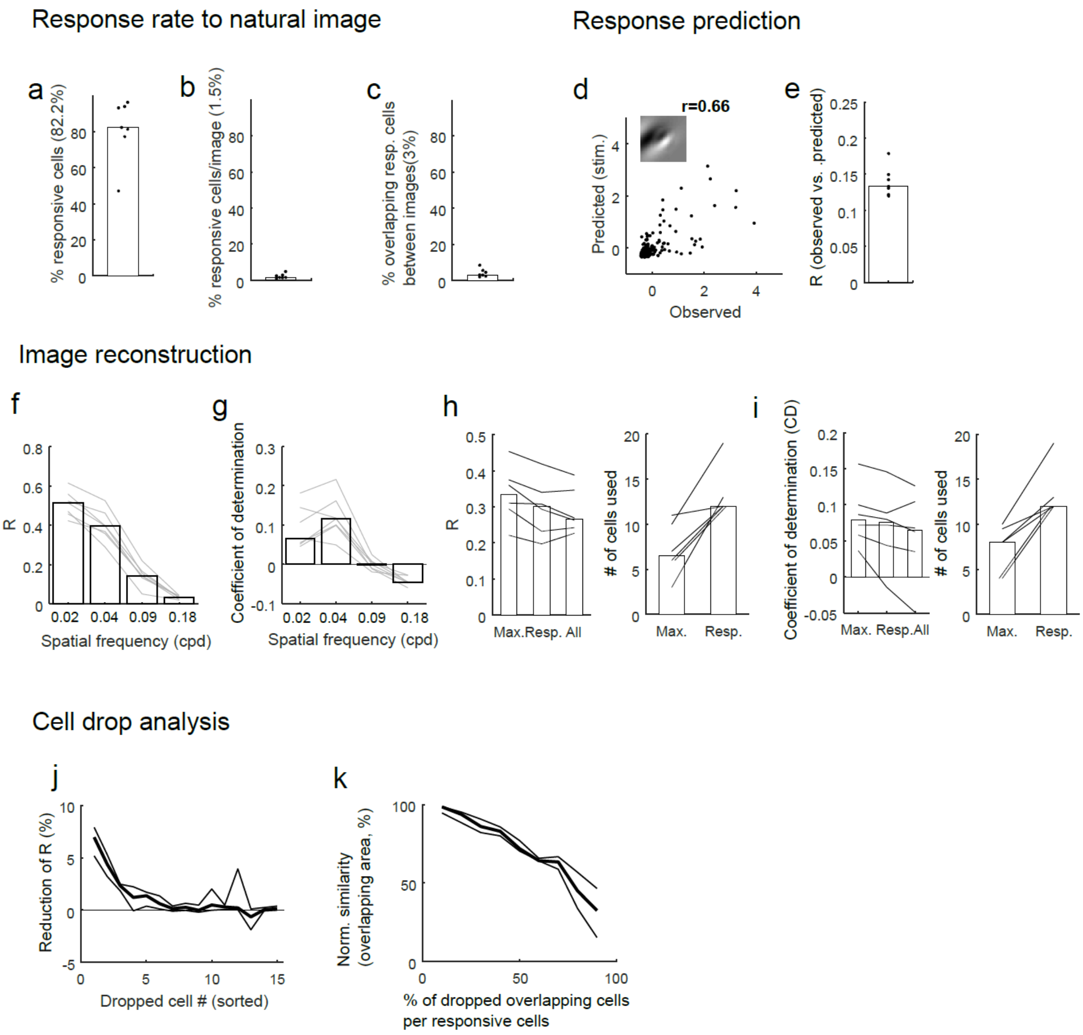

Supplementary Figure 8. Response properties, response prediction and image reconstruction in awake mice (related to Fig. 1–5)

a–**c**. Response properties (related to Fig. 1).
The percentage of responsive cells (**a**), the percentage of responsive cells in each image (**b**), and the percentage of responsive cells that overlap between two images (**c**). Most neurons in a population were visually responsive to at least one image, whereas only a small number of neurons responded to each image. Each dot indicates one plane (n = 7 planes).
d and **e**. Response prediction (related to Fig.2). (**d**) An example of the response prediction of one cell. (**e**) Response prediction performance (n = 7 planes).
f–**i**. Image reconstruction performance by the cell-selection model (related to Fig. 3).
(**f** and **g**) Reconstruction performance at each spatial frequency (**f**: R and **g**: CD).
(**h** and **i**) Left panels: Performances obtained from all cells (All), responsive cells (Resp.) and cells with peak performance (Max.) were compared. The peak performance was assessed among responsive neurons. No significant difference was obtained in any pairs (signed-rank test with Bonferroni correction). Right panels: The number of cells used for the reconstruction of each image in Resp. and Max. Peak performances were obtained from fewer cells compared to the number of responsive cells (**h**: P = 0.03; **i**: P = 0.03 by signed-rank test). In the analyses (**h** and **i**), we used only data for images

that had at least 10 responsive cells and excluded one plane of data because of a small number of samples.
j and **k**. Cell drop analysis (related to Fig. 5). (**j**) Reduction in R after dropping a single cell. The order of dropped cells was obtained using their evoked response amplitude (displayed in descending order). A single cell drop only slightly reduced the performance. (**k**) Reduction in reconstruction performance during the sequential dropping of overlapping cells. The performance was estimated using the similarity (pixel-to-pixel correlation) of the overlapping area (see Fig. 5c, Supplementary Figure 6 and Methods). The analyses in (**j** and **k**) include only data for images that had at least five responsive cells. Source data are provided as a Source Data file.

Supplementary Figure 9. Reliable image representation across trials in awake mice (related to Fig. 6).

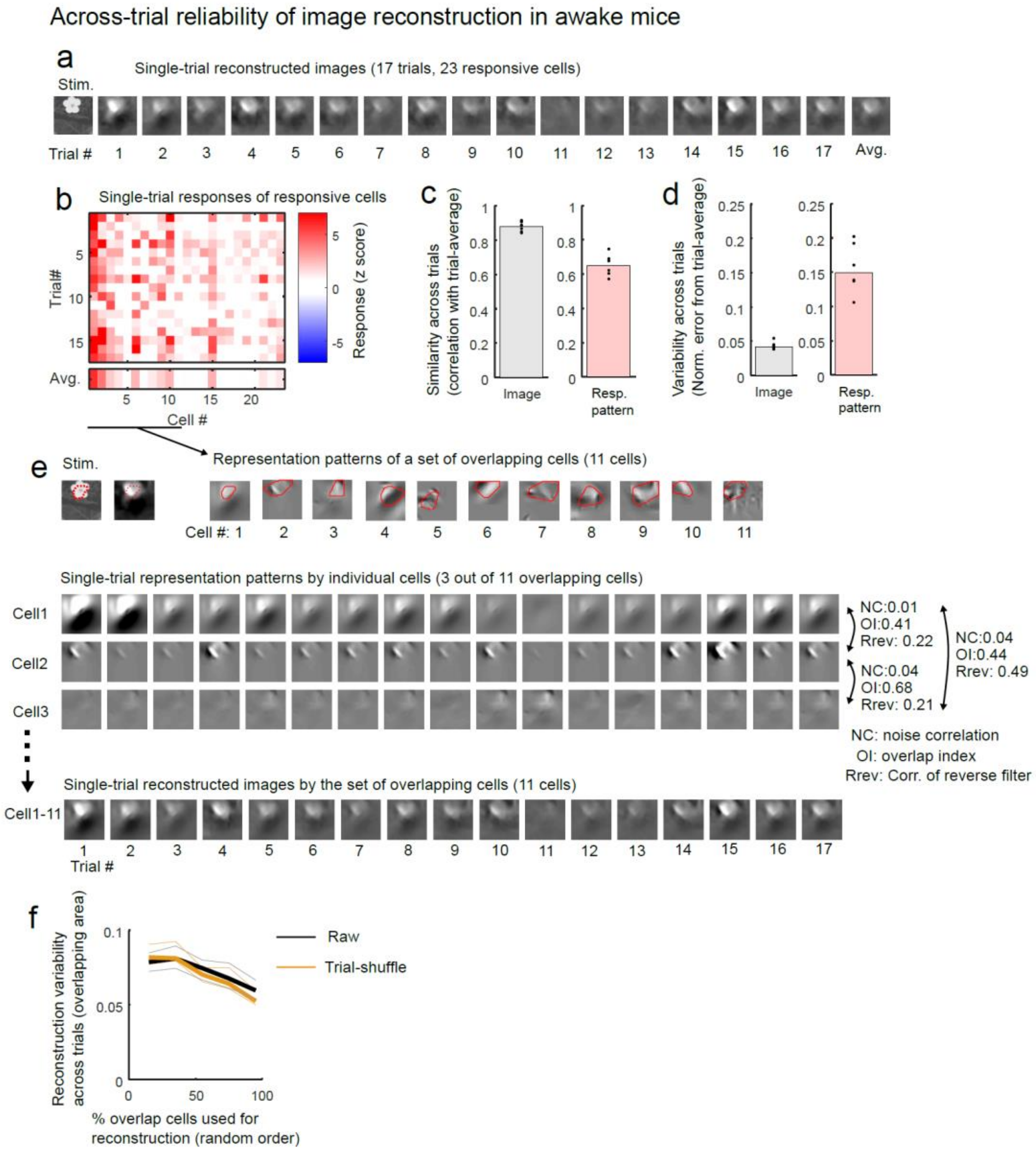

Supplementary Figure 9. Reliable image representation across trials in awake mice (related to Fig. 6).

a. Examples of single-trial reconstructed images. First panel: Stimulus image. Last panel: Trial-averaged image.
b. Single-trial evoked responses to the image in (**a**).
c. Across-trial similarity of the reconstructed image (left) and of the response patterns of the responsive cells (right). The across-trial similarity was the Pearson's correlation between a single-trial reconstructed image and the corresponding trial-averaged image (n = 6 planes).
d. Across-trial variability of reconstructed images (left) and response patterns of responsive cells (right). The normalized squared error between a single-trial image (or response pattern) and the trial-averaged image (or response pattern) was computed and averaged across trials for the across-trial variability (n = 6 planes).

e. Reconstructed image from a set of overlapping cells. Upper left panels: Stimulus and trial-averaged reconstructed images from the overlapping cells. The red dotted line indicates the overlapping area. Upper right panels: Representation (reverse filters) of the overlapping cells. The red line in each panel indicates the representation area. Cell 1 is the reference cell. Lower panels: Single-trial representations of three example cells selected from the overlapping cells. Bottom panels: Single-trial reconstructed image obtained from the overlapping cells.
f. Across-trial variability against the percentage of overlapping cells used for the reconstruction. N = 6 planes. Black lines: raw data. Orange lines: trial-shuffled data. Thick and thin lines are the median and 25th or 75th percentile of the variability among 6 planes.
In the analyses shown in this figure, we used only data for images that had at least five responsive cells and excluded one plane of data because of a small number of samples.
The stimulus images in (**a** and **e**) are adapted, with permission, from the database in supplementary reference 1. Source data are provided as a Source Data file.

Supplementary Figure 10. Image representation in excitatory and inhibitory cells.

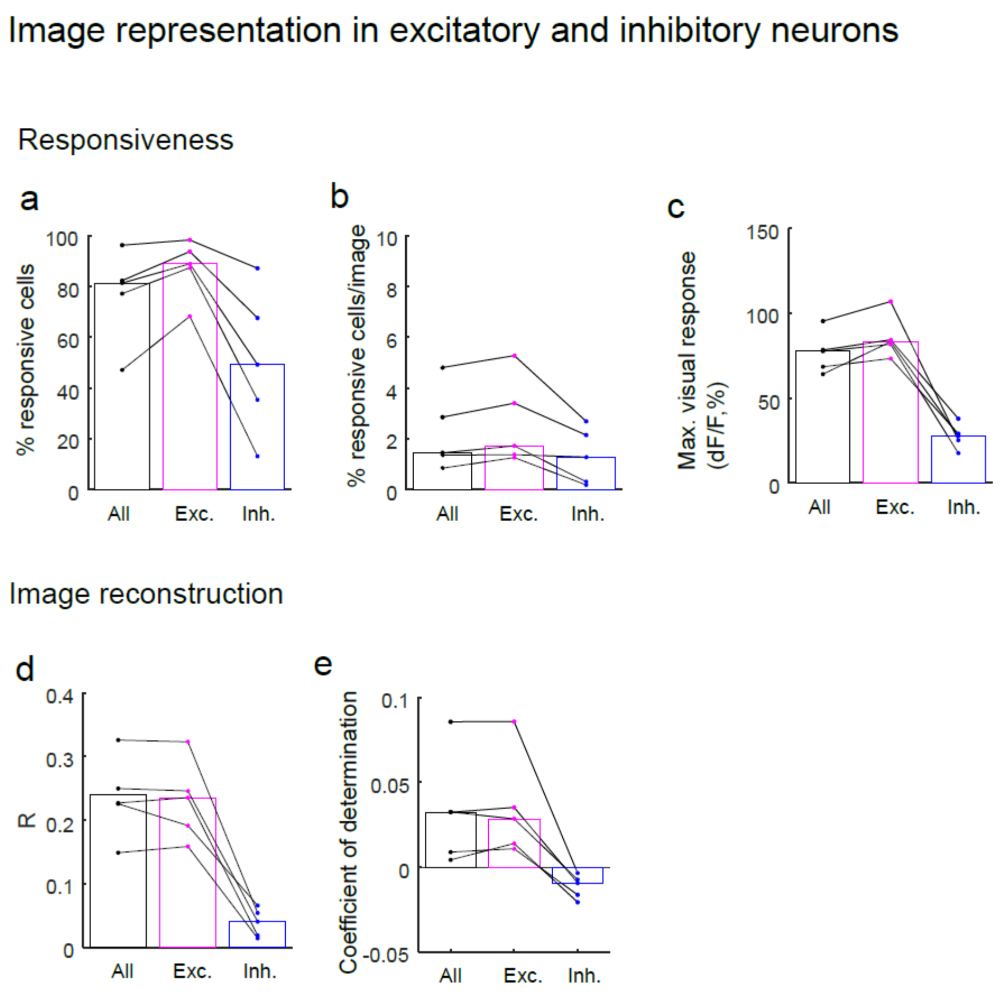

Supplementary Figure 10. Image representation in excitatory and inhibitory cells.

a–**c**. Response properties in excitatory (Exc.), inhibitory (Inh.), and all cells including both types (All). (**a**) The percentage of responsive cells. (**b**) The percentage of responsive cells for each image. (**c**) Maximal evoked response.

d and **e**. Image reconstruction performances. The performances of excitatory cells were almost comparable with those of all cells, indicating that the images are mainly represented by excitatory cells. (**a**–**e**) N = 5 planes.

Source data are provided as a Source Data file.

Supplementary Figure 11. Comparison of image reconstructions between resting and running periods

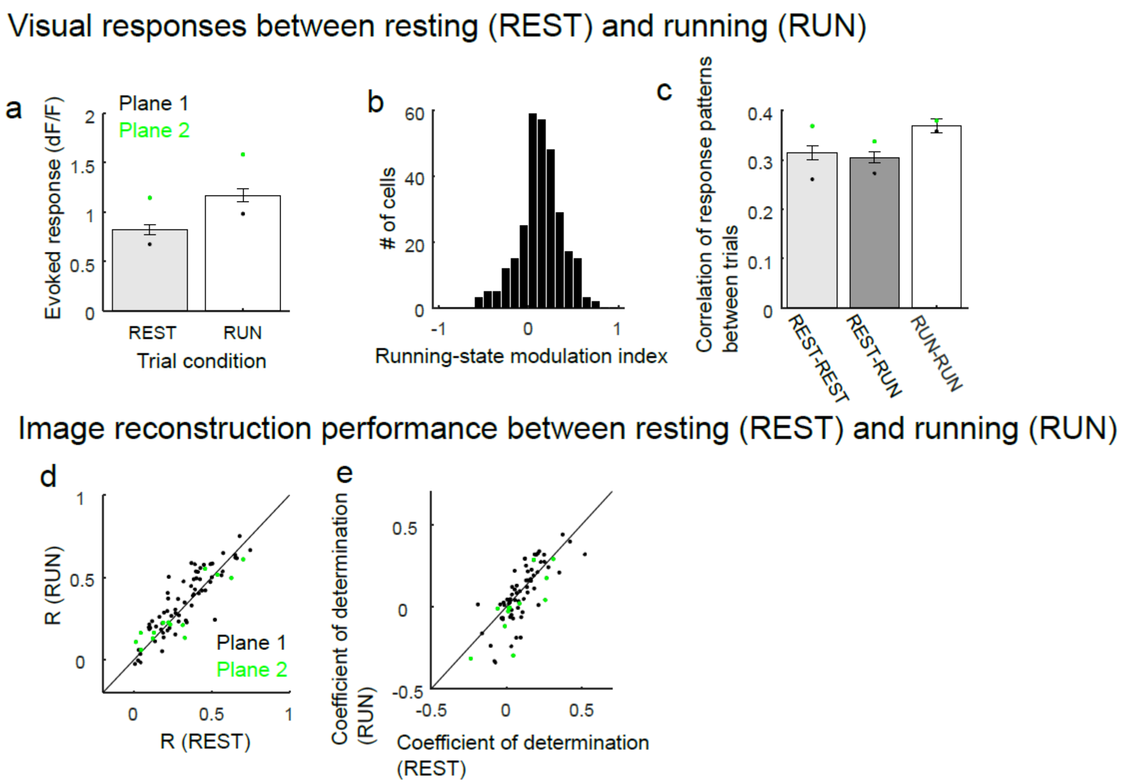

Supplementary Figure 11. Comparison of image reconstructions between resting and running periods

This analysis used only data for images that had at least 5 responsive cells and 4 trials for both resting and running states (data for 80 image cases, 295 responsive cells from two planes).
a–**c**. Comparisons of visual responses between resting and running periods. (**a**) Evoked response amplitude (n = 295 responsive cells). Bars and error bars indicate the mean and standard error of means across all cells used in this analysis. (**b**) Distribution of the running-state modulation index (n = 295 cells). (**c**) Correlation coefficients of the response patterns between trials (n = 80 image cases). Although visually evoked responses during running tended to be higher than those during resting, response patterns were similar between the conditions. (**a** and **c**) Each dot indicates the mean of each plane.
d and **e**. Comparison of image reconstruction performance between the conditions. (**d**) Pixel-to-pixel correlations between stimulus and reconstructed images (R). Each dot indicates each image case (REST: 0.31±0.005. RUN: 0.34±0.005, p = 0.02 by signed-rank test, n = 80 images). (**e**) Coefficient of determination (CD. REST: 0.08 ± 0.004. RUN: 0.03 ± 0.007, p = 0.07 by signed-rank test, n = 80 images). Different colours of dots indicate data from different planes.
Source data are provided as a Source Data file.

Supplementary References

1. van Hateren, J.H. & van der Schaaf, A. Independent component filters of natural images compared with simple cells in primary visual cortex. *Proc Biol Sci* **265**, 359-366 (1998).

2. Olmos, A. & Kingdom, F.A. A biologically inspired algorithm for the recovery of shading and reflectance images. *Perception* **33**, 1463-1473 (2004).
