# Supplementary material for: Natural images are reliably represented by sparse and variable populations of neurons in visual cortex
Source: Nat Commun. 2020 Feb 13;11:872. doi: 10.1038/s41467-020-14645-x (PMC7018721; doi:10.1038/s41467-020-14645-x)
Supplement: Supplementary file 2 — Reporting Summary [file 41467_2020_14645_MOESM2_ESM.pdf]

nature research

Corresponding author(s): Kenichi Ohki, Takashi Yoshida

Last updated by author(s): Jan 5, 2020

Reporting Summary

Nature Research wishes to improve the reproducibility of the work that we publish. This form provides structure for consistency and transparency in reporting. For further information on Nature Research policies, see Authors & Referees and the Editorial Policy Checklist .

Statistics

For all statistical analyses, confirm that the following items are present in the figure legend, table legend, main text, or Methods section.

n/a	Confirmed	
☐	☒	The exact sample size (n) for each experimental group/condition, given as a discrete number and unit of measurement
☐	☒	A statement on whether measurements were taken from distinct samples or whether the same sample was measured repeatedly
☐	☒	The statistical test(s) used AND whether they are one- or two-sided<br>*Only common tests should be described solely by name; describe more complex techniques in the Methods section.*
☒	☐	A description of all covariates tested
☐	☒	A description of any assumptions or corrections, such as tests of normality and adjustment for multiple comparisons
☐	☒	A full description of the statistical parameters including central tendency (e.g. means) or other basic estimates (e.g. regression coefficient) AND variation (e.g. standard deviation) or associated estimates of uncertainty (e.g. confidence intervals)
☐	☒	For null hypothesis testing, the test statistic (e.g. F, t, r) with confidence intervals, effect sizes, degrees of freedom and P value noted<br>*Give P values as exact values whenever suitable.*
☒	☐	For Bayesian analysis, information on the choice of priors and Markov chain Monte Carlo settings
☒	☐	For hierarchical and complex designs, identification of the appropriate level for tests and full reporting of outcomes
☐	☒	Estimates of effect sizes (e.g. Cohen's d, Pearson's r), indicating how they were calculated

Our web collection on statistics for biologists contains articles on many of the points above.

Software and code

Policy information about availability of computer code

Data collection	We used the commercially available software (NIS element, NIKON) for two-photon imaging data acquisition, Psychopy2 for visual stimulus presentation, and a custom LabView code for aquisitions of eye images and rotary encoder signals.
Data analysis	We used custom MATLAB codes for data analyses. Codes for Gabor transformation and reconstruction were based on a open-source sofware (https://visiome.neuroinf.jp/database/item/6894).

For manuscripts utilizing custom algorithms or software that are central to the research but not yet described in published literature, software must be made available to editors/reviewers. We strongly encourage code deposition in a community repository (e.g. GitHub). See the Nature Research guidelines for submitting code & software for further information.

Data

Policy information about availability of data

All manuscripts must include a data availability statement. This statement should provide the following information, where applicable:

- Accession codes, unique identifiers, or web links for publicly available datasets
- A list of figures that have associated raw data
- A description of any restrictions on data availability

The datasets of the current study and the codes used to analyse them are available from the corresponding authors upon request.

Field-specific reporting

Please select the one below that is the best fit for your research. If you are not sure, read the appropriate sections before making your selection.

☒ Life sciences ☐ Behavioural & social sciences ☐ Ecological, evolutionary & environmental sciences

Life sciences study design

All studies must disclose on these points even when the disclosure is negative.

Sample size	The sample sizes were not predetermined by any statistical methods but are comparable to the sample size of other reports in the field.
Data exclusions	In the analyses in Fig1h and 1i, data for one plane was discarded because of a large drift of the imaging plane during the recording.<br>In the analyses in Fig. 4 (and related Supplementary figures), data for stimulus images that have less than 10 responsive cells were excluded to precisely evaluate relationship between the number of cells and image reconstruction performances. In the analyses in Fig. 5 and 6 (and related Supplementary figures), data for stimulus images that have less than five responsive and overlapping cells were excluded to precisely evaluate how cell-adding or dropping affects the image reconstruction. This led to an exclusion of one plane data for awake mice. In the analyses for running effects on the image reconstruction (Supplementary fig. 12), we used only data from two planes in one mouse (n = 295 responsive cells) because only one mouse ran relatively frequently during imaging and we did not obtained enough data during both running and staying in other mice. These are described in the main manuscript.
Replication	Most results were reliably observed across planes obtained from multiple mice.
Randomization	Randomization was not relavant to our study because this study examined V1 visual processing in a single experimental condition for anaesthetized and awake mice and because the results were not directly compared between multiple experimental conditions.
Blinding	Blinding was not relavant to our study because this study examined V1 visual processing in a single experimental condition for anaesthetized and awake mice and because the results were not directly compared between multiple experimental conditions.

Reporting for specific materials, systems and methods

We require information from authors about some types of materials, experimental systems and methods used in many studies. Here, indicate whether each material, system or method listed is relevant to your study. If you are not sure if a list item applies to your research, read the appropriate section before selecting a response.

Materials & experimental systems

n/a	Involved in the study	
☒	☐	Antibodies
☒	☐	Eukaryotic cell lines
☒	☐	Palaeontology
☐	☒	Animals and other organisms
☒	☐	Human research participants
☒	☐	Clinical data

Methods

n/a	Involved in the study	
☒	☐	ChIP-seq
☒	☐	Flow cytometry
☒	☐	MRI-based neuroimaging

Animals and other organisms

Policy information about studies involving animals; ARRIVE guidelines recommended for reporting animal research

Laboratory animals	We used adult mice (2-4 month old) including both male and female of the following strains: wild-type C57BL/6 mice for OGB1 imaging, Thy1-GCaMP6s transgenic mice and transgenic mice obtained by crossing Gad2-cre and Ai14 (loxp-tdTomato) for GCaMP imaging.
Wild animals	This study did not use wild animals.
Field-collected samples	This study did not use field-collected samples.
Ethics oversight	All experimental procedures were approved by the local Animal Use and Care Committee of Kyushu University and the University of Tokyo.

Note that full information on the approval of the study protocol must also be provided in the manuscript.
